# Conservation and Diversity in Gibberellin-Mediated Transcriptional Responses Among Host Plants Forming Distinct Arbuscular Mycorrhizal Morphotypes

**DOI:** 10.3389/fpls.2021.795695

**Published:** 2021-12-16

**Authors:** Takaya Tominaga, Chihiro Miura, Yuuka Sumigawa, Yukine Hirose, Katsushi Yamaguchi, Shuji Shigenobu, Akira Mine, Hironori Kaminaka

**Affiliations:** ^1^The United Graduate School of Agricultural Sciences, Tottori University, Tottori, Japan; ^2^Faculty of Agriculture, Tottori University, Tottori, Japan; ^3^Functional Genomics Facility, NIBB Core Research Facilities, National Institute for Basic Biology, Okazaki, Japan; ^4^Laboratory of Plant Pathology, Graduate School of Agriculture, Kyoto University, Kyoto, Japan; ^5^JST, PRESTO, Kawaguchi, Japan

**Keywords:** arbuscular mycorrhizal symbiosis, comparative transcriptomics, gibberellin, arbuscular mycorrhizal morphotypes, *Lotus japonicus*, *Daucus carota*, *Eustoma grandiflorum*, *Rhizophagus irregularis*

## Abstract

Morphotypes of arbuscular mycorrhizal (AM) symbiosis, *Arum*, *Paris*, and Intermediate types, are mainly determined by host plant lineages. It was reported that the phytohormone gibberellin (GA) inhibits the establishment of *Arum*-type AM symbiosis in legume plants. In contrast, we previously reported that GA promotes the establishment of *Paris*-type AM symbiosis in *Eustoma grandiflorum*, while suppressing *Arum*-type AM symbiosis in a legume model plant, *Lotus japonicus*. This raises a hitherto unexplored possibility that GA-mediated transcriptional reprogramming during AM symbiosis is different among plant lineages as the AM morphotypes are distinct. Here, our comparative transcriptomics revealed that several symbiosis-related genes were commonly upregulated upon AM fungal colonization in *L. japonicus* (*Arum*-type), *Daucus carota* (Intermediate-type), and *E. grandiflorum* (*Paris*-type). Despite of the similarities, the fungal colonization levels and the expression of symbiosis-related genes were suppressed in *L. japonicus* and *D. carota* but were promoted in *E. grandiflorum* in the presence of GA. Moreover, exogenous GA inhibited the expression of genes involved in biosynthetic process of the pre-symbiotic signal component, strigolactone, which resulted in the reduction of its endogenous accumulation in *L. japonicus* and *E. grandiflorum*. Additionally, differential regulation of genes involved in sugar metabolism suggested that disaccharides metabolized in AM roots would be different between *L. japonicus* and *D. carota*/*E. grandiflorum*. Therefore, this study uncovered the conserved transcriptional responses during mycorrhization regardless of the distinct AM morphotype. Meanwhile, we also found diverse responses to GA among phylogenetically distant AM host plants.

## Introduction

More than 70% of terrestrial plants associate with the symbiotic, arbuscular mycorrhizal (AM) fungi that belong to Glomeromycotina (Brundrett and Tedersoo, 2018). AM fungi supply minerals, such as inorganic phosphate and nitrogen, to their host plants, thus promoting the growth of the hosts (Ezawa and Saito, 2018; Wang et al., 2020). In return, they obtain carbohydrates, such as fatty acids, lipids, and monosaccharides, from the host plants (Bravo et al., 2017; An et al., 2019). This mutual interaction is established through several steps. Host-derived signal molecules, strigolactones (SLs), are exudates into the rhizosphere to attract AM fungi prior to the mutualism (Akiyama et al., 2005; Besserer et al., 2008; Tsuzuki et al., 2016). SLs positively regulate formation of hyphopodia on the host root epidermis (Kobae et al., 2018). After AM fungal hyphae invade the host epidermis, AM fungi form highly branched hyphal structures, the arbuscule, in the root cortical cells for nutrient exchange. Some transporters are localized on a specialized plant-derived membrane, periarbuscular membrane (PAM), to influx mineral nutrients and efflux carbohydrates such as lipids and glucose between the host and fungal symbionts (Kobae and Hata, 2010; Bravo et al., 2017; Luginbuehl and Oldroyd, 2017).

The morphology of AM fungal hyphae is known to be distinct mainly depending on the host plant species (Smith and Smith, 1997; Dickson et al., 2007). *Arum*-type AM shows that AM fungal hyphae elongate in the intercellular space of the host cortex and form arbuscules in the cortical cells. This AM morphotype is found in rice (*Oryza sativa*) and legume model plant roots such as *Medicago truncatula* and *Lotus japonicus* (Hong et al., 2012; Yu et al., 2014; Takeda et al., 2015). On the other hand, in *Paris*-type AM, the fungal hyphae invade the adjacent cortical cells and show hyphal coils on which arbuscules are formed (Smith and Smith, 1997; Dickson, 2004; Dickson et al., 2007). Moreover, an “Intermediate” type of AM showing both morphological features of *Arum*- and *Paris*-type AMs is also found in some host plants (Dickson, 2004). According to Dickson (2004), Intermediate-type AM is defined by either the existence of linear intracellular hyphae on which arbuscules are formed or hyphal coils with intercellular hyphae. The linear intercellular hyphae are sometimes found with intercellular hyphae (Dickson, 2004).

Several phytohormones have been revealed to regulate AM symbiosis. For instance, exogenous treatment of gibberellin (GA) severely reduces the number of hyphopodia and disturbs the development of arbuscule (Floss et al., 2013; Yu et al., 2014; Takeda et al., 2015; Pimprikar et al., 2016). Moreover, GA represses the expressions of some AM symbiosis-related genes (Takeda et al., 2015; Pimprikar et al., 2016; Nouri et al., 2021). Notably, it has shown that a GRAS transcription factor (TF) required for AM symbiosis, *REDUCED ARBUSCULAR MYCORRHIZA1* (*RAM1*), is transcriptionally downregulated in GA-treated *L japonicus*. This is attributable to the GA-triggered degradation of GA-signaling repressor, DELLA, which positively regulates *RAM1* expression (Silverstone et al., 2001; Achard and Genschik, 2009; Floss et al., 2013; Park et al., 2015). Notably, the *RAM1* also regulates other downstream AM marker genes: *REDUCED FOR ARBUSCULE DEVELOPMENT1 (RAD1*)–GRAS TF, *Vapyrin* (*Vpy*) (protein that regulates arbuscule development), *PHOSPHATE TRANSPORTER4* (*PT4*), *AMMONIUM TRANSPORTER2;2* (*AMT2;2*), *FatM* (acyl-acyl carrier protein thioesterase), *RAM2* (glycerol-3-phosphate acyltransferase), and *STR/STR2* (ABC transporters for lipids) (Gobbato et al., 2013; Park et al., 2015; Rich et al., 2015, 2017; Pimprikar et al., 2016; Muller et al., 2020). Thus, it has been thought that exogenous GA or the absence of functional DELLA attenuates the transcriptional promotion of downstream genes to inhibit AM fungal colonization. Interestingly, our previous study showed that GA suppresses *Arum*-type AM symbiosis in *L. japonicus* and chive, whereas promoting *Paris*-type AM symbiosis in *Eustoma grandiflorum* and *Primula malacoides* (Tominaga et al., 2020a). Another expression analysis also revealed that the expression levels of AM symbiosis-related genes in *E. grandiflorum* were increased by GA (Tominaga et al., 2020b). These findings let us hypothesize that the regulatory mechanisms underlying AM symbiosis would be diverse among host plants; however, our past studies did not simultaneously compare the GA-mediated transcriptional regulation among various host plants. To date, the effect of GA on Intermediate-type AM symbiosis has not been investigated yet.

In this study, we conducted comparative transcriptomics among three AM host plants with different AM morphotypes: *L. japonicus* (*Arum*-type AM), *E. grandiflorum* (*Paris*-type AM), and *Daucus carota* (Intermediate-type AM) (Dickson, 2004). Based on plastid genomes, Fabales, Gentianales, and Apiales, to which *L. japonicus*, *D. carota*, and *E. grandiflorum* belong, are estimated to appear *c*. 100 Ma, *c*. 80 Ma, and *c*. 90 Ma, respectively (Li et al., 2019). Our study revealed that *Rhizophagus irregularis* infection promoted shoot growth and the expression of several symbiosis-related genes conserved in all examined plants, such as *RAM1* and *STR*. However, the AM fungus-promoted expression of the conserved symbiosis-related genes was decreased in GA-treated *L. japonicus* (*Arum*-type) and *D. carota* (Intermediate-type). In contrast, the expression levels of the conserved genes were not reduced but rather increased by exogenous GA in *E. grandiflorum* (*Paris*-type). This suggests that the transcriptional reprogramming associated with AM symbiosis in *E. grandiflorum* would be tolerant to GA and unique to this plant species. Additionally, the negative effects of GA on SL biosynthetic process were commonly observed in *L. japonicus* and *E. grandiflorum*, suggesting that GA-promoted fungal colonization in *E. grandiflorum* is independent of SLs. Thus, our study uncovered the conserved responses of phylogenetically distant AM host plans regardless of AM morphotypes. Furthermore, our findings help understand the diverse effects of GA on host plant species.

## Materials and Methods

### Growth Condition of Plant and Fungal Materials

The seedlings of *L. japonicus* “Miyakojima” MG-20, *D. carota* cv. Nantes, and *E. grandiflorum* cv. Pink Thumb were prepared as in our previous report (Tominaga et al., 2020a). *D. carota* seedlings were grown in light for 7 days. Since *E. grandiflorum* exhibited relatively low colonization rates in our previous report (Tominaga et al., 2020a), high concentration of AM fungal spores, approximately 6,000 spores of *R. irregularis* DAOM197198 (Premier Tech, Quebec, Canada), were added to 50 ml 1/5 Hoagland solution containing 20 μM inorganic phosphate. GA_3_ was dissolved in ethanol and treated at this procedure by diluting the stock to the 1/5 Hoagland solution at 1 μM. Ethanol was treated in the same way as the control treatment. The solution was added to approximately 300 ml autoclaved mixed soil (river sand/vermiculite, 1:1) in a plastic container combined with another one as described in Takeda et al. (2015). As a result, each tested seedling was inoculated with 1,000 spores of *R. irregularis*. Then, the prepared seedlings were transplanted into the soil and kept for 6 weeks under 14 h light/10 h dark cycles at 25°C.

### Quantification and Observation of Arbuscular Mycorrhizal Symbiosis

The inoculated roots were harvested at 6 weeks post-inoculation (wpi), and fixation, staining, and quantification of AM fungal colonization rates were conducted according to previous studies (Mcgonigle et al., 1990; Tominaga et al., 2020a). To determine the AM morphotypes of root samples stained with trypan blue, single cortex layer containing AM fungal hyphae was microscopically observed by gently squashing the roots.

For fluorescence images, the fragments of fixed roots were rinsed with phosphate-buffered saline (PBS) and immersed in ClearSee (FUJIFLIM Wako Pure Chemical, Osaka, Japan) for 1 week in the dark (Kurihara et al., 2015). The instructions of the manufacturer were followed in the clearing procedure. The cleared roots were rinsed with PBS and stained with 0.01 mg/ml WGA-Alexa Fluor 488 (Thermo Fisher Scientific, Waltham, MA, United States) for 15 min. For the staining of plant cell wall, the root samples were further treated with 20 μg/ml Calcofluor White (Sigma-Aldrich, St Louis, MO, United States) for 15 min. Under a fluorescent stereomicroscope, Leica M205 FCA (Leica Microsystems, Wetzlar, Germany), the relatively bright fluorescent region, which indicates colonized area, was excised with a scalpel and embedded in 5% (w/v) agarose containing 1% (w/v) gelatin. Then, 30–50 μm cross sections were made using a Linear Slicer PRO-7 (Dosaka EM, Kyoto, Japan) and observed under a FLUOVIEW FV10i confocal laser scanning microscope (Olympus, Tokyo, Japan) with 499 nm excitation and 520 nm emission for WGA-Alexa Fluor 488 and FV10i-SW software v1.2 (Olympus, Tokyo, Japan). The images were merged using the ImageJ software v1.51k^1^.

### Transcriptome Analysis

#### RNA Extraction and RNA Sequencing

Root samples (approximately 100 mg) at 6 wpi were collected in a nuclease-free tube (INA-OPTIKA, Osaka, Japan) with two 5 mm beads, frozen by liquid nitrogen. The frozen root samples were set in ShakeMan6 (Bio-Medical Science, Tokyo, Japan) and homogenized. Then, the total RNA was extracted using the real RNA Extraction Kit Mini for Plants (RBC Bioscience, New Taipei, Taiwan) following the protocol of the manufacturer. RNase-free DNase I (Takara Bio, Shiga, Japan) was applied to remove genomic DNA from the RNA samples according to the instructions of the manufacturer. The purity and quantity of the total RNA was calculated by measuring the absorbance at 260 and 280 nm (*A*260: *A*280) with DeNovix DS-11+ (Scrum, Tokyo, Japan). RNA-seq library was constructed from the total extracted RNA and sequenced, and RNA-seq with strand-specific and paired-end reads (150 bp) was performed with DNBSEQ-G400 by Genewiz (Tokyo, Japan). Consequently, more than 20 million raw reads per sample were obtained (Supplementary Table 1). Low-quality reads (<QV30) and adapter sequences were removed by Fastp (Chen et al., 2018).

#### Data Analysis

Read mapping was conducted using STAR (Dobin et al., 2013) for the filtered single-end reads of *L. japonicus*, *D. carota*, and *R. irregularis* onto their genomes, Lotus japonicus Lj1.0v1, Daucus carota v2.0, and Rir_HGAP_ii_V2, retrieved from the Phytozome v13^2^ and Ensembl Fungi^3^ (Iorizzo et al., 2016; Maeda et al., 2018; Li et al., 2020). Meanwhile, Bowtie2 with default parameters except for “–loc al” was applied for *E. grandiflorum* to map the reads to *de novo* reference assembly constructed from previous RNA-seq data (Tominaga et al., 2020b) by Trinity v2.8.4 (Grabherr et al., 2011; Langmead and Salzberg, 2012; Haas et al., 2013). In this study, we mapped the reverse reads to the indicated genomes or *de novo* assembly data to perform specific alignment. The number of mapped reads to the reference genome was counted using featureCounts v1.6.4 (Liao et al., 2014) for *L. japonicus*, *D. carota*, and *R. irregularis*, whereas that of *E. grandiflorum* was quantified with eXpress v1.5.1 (Roberts and Pachter, 2013) due to using *de novo* assembled cDNA sequences as reference unlike others. For identifying differentially expressed genes (DEGs), each count data showing different library sizes were normalized by the trimmed mean of the *M*-values normalization method, and genes with | Log_2_ fold change (FC)| > 1 and false discovery rate (FDR) less than the indicated values (FDR < 0.01 for plants’ DEGs and FDR < 0.05 for fungal DEGs) were considered DEGs using the EdgeR package (Robinson et al., 2010).

The transcripts per million (TPM) (Li et al., 2010; Wagner et al., 2012) of each sample was counted from the count data using the R software v4.0.2 (R Foundation for Statistical Computing). Genes that showed zero counts in all samples were excluded from the analysis, unless otherwise noted. Then, the mean TPM of all samples in a condition was Log_2_-transformed for each gene. The heatmaps in this study were constructed using the heatmaply package in R (Galili et al., 2018).

#### Gene Ontology Enrichment Analysis

Differentially expressed gene was sorted depending on their expression patterns using a Venn diagram^4^. Then, the gene ontology (GO) enrichment analysis was conducted using the ClueGO plugin for Cytoscape (Bindea et al., 2009, 2013). Additionally, the correlation network of enriched GO terms was created using the ClueGO. In the analysis, *p*-values were calculated using a two-sided hypergeometric test and corrected using the Benjamini–Hochberg method. The GO terms of *R. irregularis* were annotated by EnTAP v0.10.7 (Hart et al., 2020), followed by GO enrichment analysis using the topGO package in the R environment. In the topGO study, the enrichment test was performed by calculating the *p*-values using the Fisher’s exact test (*p* < 0.01) and scoring with the *Elim* method (Alexa et al., 2006). The *p*-values of filtered GO terms were adjusted by the Benjamini–Hochberg method.

### Ortholog Identification

Here, we identified ortholog genes in *L. japonicus*, *D. carota*, and *E. grandiflorum* to compare the influence of AM fungal colonization and GA treatment among these different host species. The proteomes of *L. japonicus* and *D. carota* were retrieved from the Phytozome v12.1 and v13, respectively. Additionally, coding sequence and amino acid sequences in the *de novo* assembly of *E. grandiflorum* were predicted using TransDecoder v5.5.0 (Haas et al., 2013). Next, the ortholog was identified using SonicParanoid with default parameters in the Python v3.8 environment (Cosentino and Iwasaki, 2019). Several known genes were used as queries for BLASTp search against *L. japonicus* proteome on the website Phytozome v13 (Supplementary Tables 1, 2). The resulting top hit *L. japonicus* gene and its corresponding orthologs in *D. carota* and *E. grandiflorum* were considered orthogroups and analyzed.

### Extraction of Endogenous Strigolactones and Germination Assay

To extract SLs from the host roots, we referred to the methods in a previous study with some modifications (Floková et al., 2020). The fresh 6-week-old roots (100 mg) were homogenized in ShakeMan6 (Bio-Medical Science, Tokyo, Japan) with 1 ml of 60% (v/v) acetone stored at −30°C. The suspensions were collected by centrifugation and evaporated *in vacuo* for 30 min using Savant SpeedVac DNA130 (Thermo Fisher Scientific, Waltham, MA, United States). Hydrophobic components in residual water (*c*. 500 μl) were extracted by ethyl acetate three times, and the organic layer was evaporated *in vacuo*. The samples were resolved in acetone at 400 mg FW root/ml and stored at 4°C until use. Root exudates of 4-week-old *E. grandiflorum* were collected as our previous study and rinsed with 25% acetone before elution (Tominaga et al., 2020a).

*Orobanche minor* seeds were incubated on two moist filter papers for 10 days at 24°C in the dark. An aliquot of acetone, 1 μM *rac*-GR24 (StrigoLab, Torino, Italy), and extracted samples (20 μl) were added to 6-mm glass fiber disks. Then, the conditioned *O. minor* seeds were placed on the disks with 20 μl distilled water. After 5 days of incubation at 24°C in the dark, the germination rate (%) was counted.

### Biological Replicate and Statistical Analysis

One glass slide with 10 pieces of root fragments collected from one plant was considered a biological replicate for colonization rate quantification. One glass fiber disk with *O. minor* seeds was equivalent to one biological replicate. These experiments were reproduced three times with more than five biological replicates. In the transcriptome analysis, one library constructed from a pool of total RNA consisting of three plants was treated as one biological replicates. Statistical analyses were conducted using the R software v4.0.2.

## Results

### Phenotypes of Arbuscular Mycorrhizal Roots in Different Host Plant Species Associated With *Rhizophagus irregularis*

**FIGURE 1:**
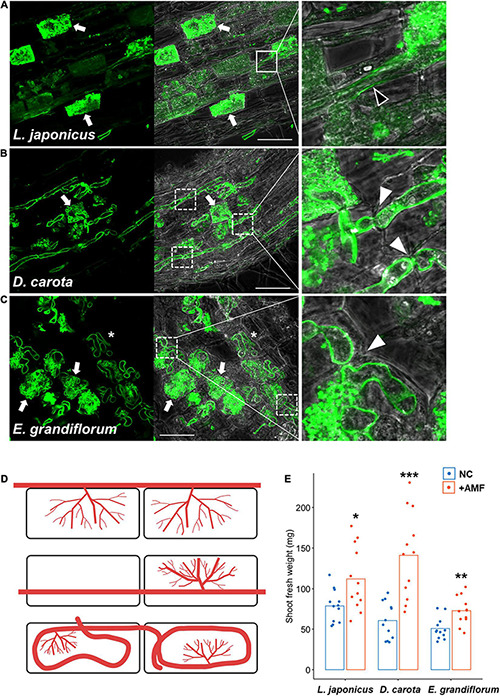
Observation of arbuscular mycorrhizal (AM) morphotypes and quantification of plant growth promotion. AM symbiosis-related phenotypes and shoot fresh weight of *Lotus japonicus*, *Daucus carota*, and *Eustoma grandiflorum* colonized by *Rhizophagus irregularis* were observed and evaluated at 6 wpi. The collected AM roots were stained with WGA-Alexa Fluor 488 (green). **(A–C)** Confocal images of *L. japonicus* **(A)**, *D. carota* **(B)**, and *E. grandiflorum* **(C)** inoculated with *R. irregularis*. The left sides of each confocal fluorescence image are merged with their images in bright field (middle). The enlarged images showing where intraradical hyphae elongate are set on the right side of the merged pictures. Arrows, arbuscules; solid-line square, intercellular hypha (black arrowhead in the enlarged image); dotted-line squares, intracellular hyphae penetrating two adjacent cortical cells (white arrowheads in the magnified images); asterisks, hyphal coils in the root cortical cells; bars, 50 μm. **(D)** Diagrams of AM morphotypes observed in *L. japonicus* (upper), *D. carota* (middle), and *E. grandiflorum* (bottom). Red lines indicate intraradical *R. irregularis* hyphae. **(E)** The shoot fresh weight (mg) of each host plant grown in axenic conditions (NC) and colonized by *R. irregularis* (+AMF) (*n* = 12). The average percentage values are shown as bars. Asterisks indicate significant differences compared with the controls in the Wilcoxon rank-sum test (*p < 0.05, **p < 0.01, ***p < 0.001).

In *L. japonicus*, a typical *Arum*-type AM with intercellular hyphae and highly branched arbuscules in the cortical cells were formed at 6 wpi with *R. irregularis* (Figures 1A,D and Supplementary Figure 1A). We also observed *D. carota* AM roots and found linear intraradical hyphae invading the cortical cells, but we could not confirm intercellular hyphae and clear hyphal coil (Figures 1B,D and Supplementary Figures 1C,D). AM morphotype we found in *D. carota* roots is described as Intermediate 2 (I2) of four Intermediate type AMs, and *D. carota* roots associating with another AM fungus, *Glomus mosseae*, is reported to form I2 morphotype (Dickson, 2004). Therefore, we defined the AM morphotype of *D. carota* with *R. irregularis* as Intermediate-type AM in this study. On the other hand, *E. grandiflorum* showed a classical *Paris*-type AM that forms hyphal coils elongating in a circle and invading the adjacent cortical cells and an arbuscule emerging from a hyphal coil (Figures 1C,D and Supplementary Figure 1E; Tominaga et al., 2020a). Taken together, the host plant species formed distinct AM morphologies with a single fungal species, *R. irregularis*. By contrast to the distinct AM morphotypes, the shoot growth promotion by AM fungal colonization was commonly occurred in each host plant (Figure 1E). However, some exceptions, such as tomato forming both *Arum*- and *Paris*-type AMs depending on the AM fungal traits, were reported (Cavagnaro et al., 2001; Dickson, 2004; Smith et al., 2004; Kubota et al., 2005; Hong et al., 2012).

**FIGURE 2:**
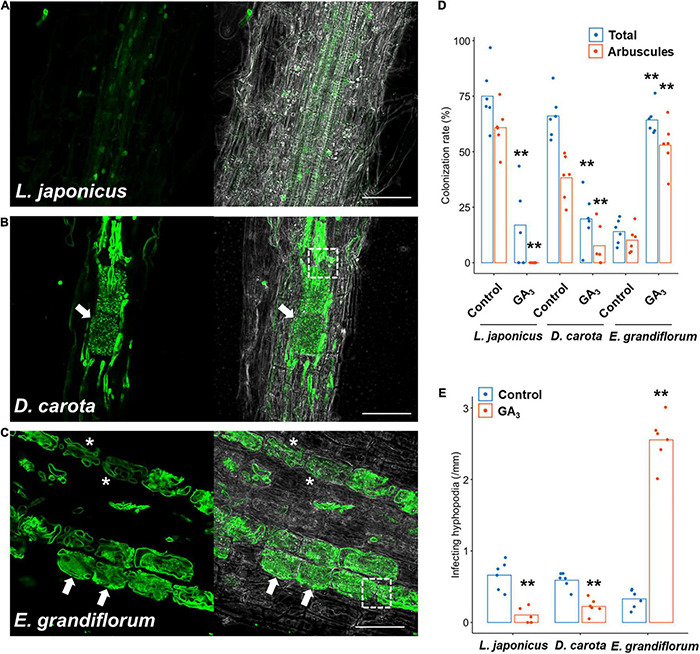
Effects of GA on hyphal structures and AM fungal colonization in tested host plants. Each host plant was treated with 0.01% ethanol (+AMF) for the control and 1 μM GA_3_ (+AMF +GA_3_) for 6 weeks. The AM root samples were stained with WGA-Alexa Fluor 488 (green). **(A–C)** Confocal images of *Lotus japonicus* **(A)**, *Daucus carota* **(B)**, and *Eustoma grandiflorum* **(C)** inoculated with *Rhizophagus irregularis* in the presence of GA. Arrows, arbuscules; dotted-line squares, intracellular hyphae invading two adjacent cortical cells, asterisks, hyphal coils; bars, 200 μm in **(A)** and 50 μm in **(B,C)**. **(D)** Quantification of AM fungal colonization rates (%) of the examined plants at 6 wpi. Total, total colonization rate; arbuscules, percentage of arbuscule formation (*n* = 5, 6). **(E)** The number of hyphopodia penetrating the host root epidermis per root length (mm) (*n* = 5, 6). The average percentage values are represented as bars. Asterisks indicate statistical significance compared with the controls in the Wilcoxon rank-sum test (**p < 0.01). AM, arbuscular mycorrhizal; GA, gibberellin.

Although we previously reported that exogenous GA treatment inhibits or promotes the establishment of *Arum*- and *Paris*-type AM symbiosis, respectively (Tominaga et al., 2020a), the effects of GA on Intermediate-type AM symbiosis remain to be cleared. Thus, we treated *L. japonicus*, *D. carota*, and *E. grandiflorum* with 1 μM GA_3_ and observed and quantified fungal colonization. In *L. japonicus* roots, we confirmed that GA treatment significantly inhibited the AM fungal colonization and arbuscule formation compared with the control AM roots, but some intercellular hyphae were still found as described in several studies (Figures 2A,D and Supplementary Figure 1B; Floss et al., 2013; Takeda et al., 2015; Pimprikar et al., 2016; Nouri et al., 2021). Interestingly, the morphologies of hyphal structures in *D. carota* and *E. grandiflorum* were not influenced by GA treatment (Figures 2B,C). Nevertheless, GA-treated *D. carota* showed reduced AM fungal colonization compared with the control (Figure 2D). These results indicate that *D. carota* can form normal but less arbuscules in the presence of GA compared with the control roots, implying that AM symbiosis in *L. japonicus* was more vulnerable to 1 μM GA_3_ than GA-suppressed AM symbiosis in *D. carota*. Additionally, GA-treated *E. grandiflorum* roots showed enhanced AM fungal infection with fully developed arbuscules at 6 wpi (Figures 2C,D) as our previous study has reported the same result at 4 wpi (Tominaga et al., 2020a). The number of AM fungal entries was consistent with the colonization rates (Figure 2E). Therefore, the association with *R. irregularis* contributed to the growth promotion in each tested plant regardless of AM morphotypes, whereas the responses to exogenous GA in *E. grandiflorum* AM roots were unique.

### Comparisons of Symbiosis-Related Genes Shed Light on Conserved and Specific Transcriptional Responses Among Arbuscular Mycorrhizal Host Plants

Based on the results in Figures 1, 2, the transcriptional regulation of downstream genes required for AM symbiosis would be expected to be different among the examined plants. To test this hypothesis, the expression pattern of genes conserved among the host plants was compared. First, orthogroups, including each known AM symbiosis-related gene, were identified using the SonicParanoid software (Supplementary Figure 2A and Supplementary Tables 2, 3).

We focused on several genes involved in AM symbiosis: *RAM1*, *RAD1*, *Vpy*, *PT4*, *AMT2;2, AMT2;3*, *FatM*, *RAM2*, *STR*, and *STR2* (Supplementary Figure 2A and Supplementary Table 3). The AP2-EREBP domain TFs, *CBX1* and *WRI5a/b/c* that are involved in fatty acid biosynthesis and reciprocally regulate the expression of *RAM1*, were also included in the analysis (Luginbuehl et al., 2017; Jiang Y. et al., 2018; Xue et al., 2018; Shi et al., 2021). Additionally, we identified the sucrose synthase 1 (*SucS1*) and glucose transporter (Sugar Will Eventually be Exported Transporter 1b; *SWEET1b*) conserved in the examined plants. In arbuscule-containing cortical cells, *SucS1* and *SWEET1b* are predicted to catalyze sucrose into glucose and export the monosaccharide *via* PAM in *M. truncatula*, respectively (Hohnjec et al., 2003; Baier et al., 2010; An et al., 2019). Notably, these genes have been reported to be transcriptionally upregulated upon AM fungal colonization.

**FIGURE 3:**
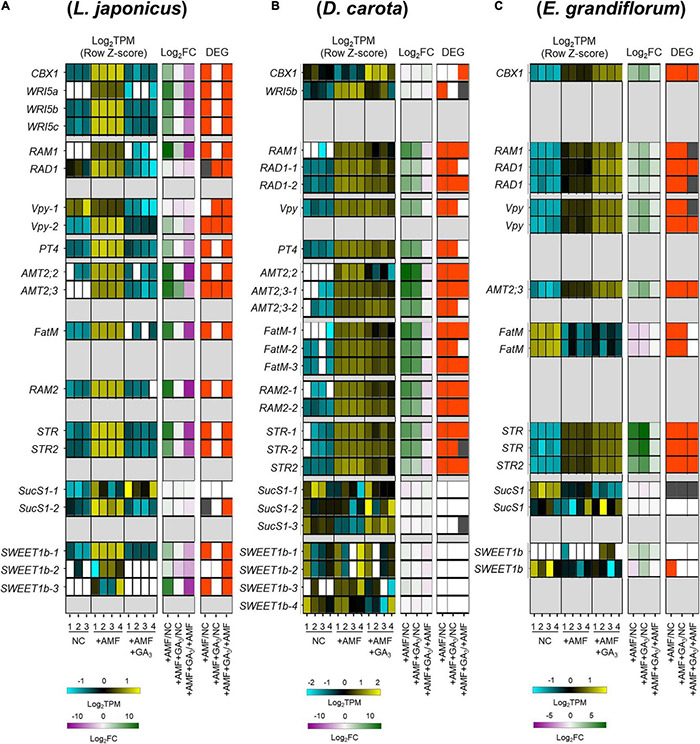
Comparative analysis on the expression patterns of AM symbiosis-related downstream genes conserved among examined host plants. **(A–C)** Heatmaps represent the expression patterns of the selected genes in response to AM fungal colonization and GA treatment in *Lotus japonicus* **(A)**, *Daucus carota* **(B)**, and *Eustoma grandiflorum* **(C)** at 6 wpi. The left heatmaps indicate the expression levels of selected genes. The Log_2_-transformed TPM in every sample is shown in blue (low expression level), black (mean), and yellow (high expression level); the expression levels are *Z*-score-normalized to turn the average value and SD to 0 and 1, respectively, across all samples. The number below the heatmaps indicates biological replicate. The middle ones show Log_2_-transformed FCs in the genes compared with the controls. Magenta indicates negative values, green represents positive values, and white means 0. The right ones illustrate significance in the fold changes in gene expression levels. DEGs (| Log_2_FC| > 1, FDR < 0.01) and genes showing significantly but slightly different expression levels compared with the controls (FDR < 0.05) are colored with red and gray, respectively. NC, non-colonized control; +AMF, *Rhizophagus irregularis* inoculation; +AMF +GA_3_, simultaneous application of *R. irregularis* inoculation and 1 μM GA_3_. The DEGs were identified by comparing +AMF with NC (+AMF/NC), +AMF +GA_3_ against NC or +AMF (+AMF +GA_3_/NC, +AMF +GA_3_/+AMF). For the TPM, Log_2_FC, and FDR values of the selected gene, see Supplementary Table 4. AM, arbuscular mycorrhizal; DEG, differentially expressed gene; FC, fold change; FDR, false discovery rate; GA, gibberellin; TPM, transcripts per million.

In this analysis, the examined plants were grown under several conditions as follows: non-colonized control roots (NC), AM roots (+AMF), and GA-treated AM roots (+AMF +GA_3_). A common set of selected genes were transcriptionally promoted upon fungal colonization at 6 wpi in each plant (Figure 3). AM fungal colonization, however, did not induce the expression of *E. grandiflorum FatM* (*EgFatM*), *D. carota*, and *E. grandiflorum SucS1*s and *SWEET1b*s at 6 wpi (Figures 3B,C). In addition to *EgFatM*, several transcripts annotated as palmitoyl-acyl carrier protein thioesterase were transcriptionally activated upon the AM fungal colonization (Supplementary Table 4). In *L. japonicus*, the expression levels of several conserved genes were undetectable or mostly reduced by exogenous GA compared with NC and +AMF conditions (Figure 3A). In contrast, the expression levels of AM symbiosis-related genes in GA-treated *D. carota* were still increased compared with the NC but decreased compared with the +AMF (Figure 3B). This suggests that the sensitivity of *D. carota* to negative effect of GA on the expression of AM symbiosis genes would be relatively moderate to that in *L. japonicus* as the colonization rates showed (Figure 2D). In *E. grandiflorum*, the expression of the AM-induced genes was further enhanced by GA than the NC and +AMF controls (Figure 3C). This result further supports the positive effect of GA on AM colonization in *E. grandiflorum* (Figure 2D).

*Eustoma grandiflorum PT4* (*EgPT4*) and *RAM2* (*EgRAM2*) were not identified by the SonicParanoid. Therefore, we conducted a BLAST search for the two genes in *E. grandiflorum* with sufficient *E*-value (<1E-5) (Supplementary Table 3). Consequently, one gene annotated as phosphate transporter (TRINITY_DN34977_c0_g1_i1.p1) was found to be homologs to *M. truncatula PT4* (Supplementary Table 3) and was transcriptionally enhanced upon the AM fungal colonization and exogenous GA (Supplementary Table 4; Tominaga et al., 2020b). Additionally, several *E. grandiflorum* genes were annotated as glycerol-3-phosphate acyltransferase (*RAM2*) (Supplementary Table 4). However, their expression levels were not promoted upon the AM fungal colonization (Supplementary Table 4). Alternatively, we might have missed *EgRAM2* in the *de novo* assembly after removing redundant contigs with CD-HIT (Tominaga et al., 2020b).

### Genes Involved in Phytohormone Biosynthesis and Signaling Show Similar Transcriptional Responses to Exogenous Gibberellin in the Examined Host Plants

**FIGURE 4:**
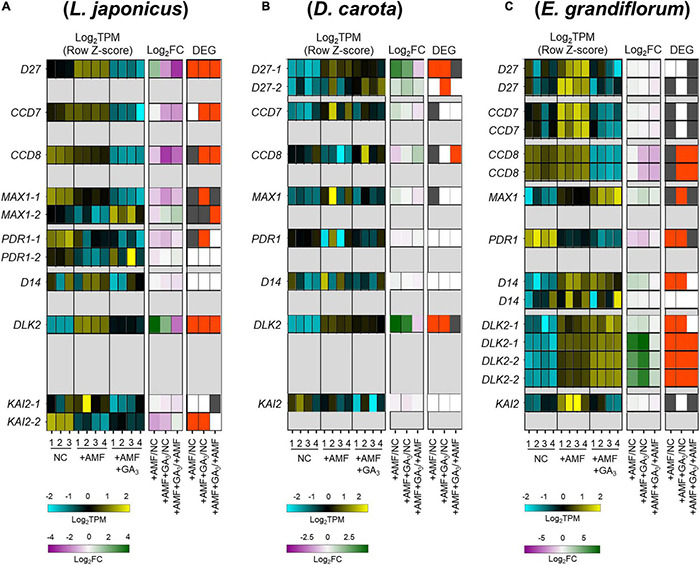
Comparative analysis of the expression patterns of SL biosynthetic and signaling-related genes conserved among examined host plants. **(A–C)** Heatmaps represent the expression patterns of the selected SL-related genes in *Lotus japonicus* **(A)**, *Daucus carota* **(B)**, and *Eustoma grandiflorum* **(C)** at 6 wpi. The expression levels and Log_2_FC compared with the controls of each gene are represented in the left heatmap (blue, low expression level; black, mean; yellow, high expression level) and the middle one (magenta, negative values; green, positive values; white, zero), respectively. The number below the heatmaps indicates a biological replicate. In the right one, the red and gray represent DEGs (| Log_2_FC| > 1, FDR < 0.01) and genes with FDR < 0.05. NC, non-colonized control; +AMF, *Rhizophagus irregularis* inoculation; +AMF +GA_3_, simultaneous application of *R. irregularis* inoculation and 1 μM GA_3_. The DEGs were identified by comparing +AMF with NC (+AMF/NC), +AMF +GA_3_ against NC or +AMF (+AMF +GA_3_/NC, +AMF +GA_3_/+AMF). The calculated values of TPM, Log_2_FC, and FDR of the selected genes can be found in Supplementary Table 4. AM, arbuscular mycorrhizal; DEG, differentially expressed gene; FC, fold change; FDR, false discovery rate; GA, gibberellin; SL, strigolactone; TPM, transcripts per million.

Since the number of infecting hyphopodia differed between *L. japonicus*/*D. carota* and *E. grandiflorum*, we also analyzed the expression patterns of several SL-related genes. *D27*, *CCD7*, *CCD8*, and *MAX1* are necessary for SL biosynthesis (Booker et al., 2004, 2005; Auldridge et al., 2006; Alder et al., 2012; Waters et al., 2012a; Al-Babili and Bouwmeester, 2015). *PDR1* in *Petunia hybrida* encoding a G-type ABC transporter is predicted to export SLs (Kretzschmar et al., 2012). Additionally, *D14*, *DLK2*, and *KAI2*, which belong to a D14 family, have previously been demonstrated as components in SL, karrikin (KAR), or KAI2-ligand (KL) signaling or both (Waters et al., 2012b; Kameoka and Kyozuka, 2015; Vegh et al., 2017). Based on the identification using SonicParanoid, these genes seemed to be conserved in *L. japonicus*, *D. carota*, and *E. grandiflorum* (Supplementary Figure 2B and Supplementary Table 3). For SL biosynthetic genes, *LjD27*, *LjCCD7*, and *LjCCD8* were transcriptionally downregulated by GA treatment (Figure 4A). Additionally, the expression of *CCD8* in *E. grandiflorum* was significantly reduced upon GA treatment, and *EgD27* and *EgCCD7* expressions were slightly inhibited by the treatment. This result suggests that GA inhibits SL biosynthesis and exudation to the rhizosphere in *E. grandiflorum* as found in *L. japonicus* and *O. sativa* (Figure 4C; Ito et al., 2017).

However, a statistically significant induction of *DcCCD8* at 2.46-fold was detected in GA-treated AM roots compared with that in the +AMF condition (Figure 4B and Supplementary Table 4). To confirm the effect of GA on SL production, we conducted a germination assay by using *O. minor* whose germination is induced by SLs (Ueno et al., 2014; Trabelsi et al., 2017). To prepare root extraction in the same conditions as the RNA-seq experiments, the examined plants were grown in the soil mixture for 6 weeks. Consistent with the expression analysis, the germination activity of root extracts was significantly reduced in *L. japonicus* by GA treatment, whereas it increased in GA-treated *D. carota* (Supplementary Figure 3). The seed germination of *O. minor* was not promoted by *E. grandiflorum* root extracts, which might be attributed to the low quantities of SLs (Sato et al., 2003; Halouzka et al., 2020). When we hydroponically grow *E. grandiflorum*, the root exudates exhibited germination activity and negative effect of GA on SL production (Supplementary Figure 3C). Taken together, the enhanced AM fungal colonization in GA-treated *E. grandiflorum* would be mainly supported by unidentified components but not by SLs.

Of the SL signaling-related genes, *DLK2* was significantly induced in the examined host plants upon AM fungal colonization compared with NC control (Figure 4). Similarly, *DLK2* induction was reported in other host plants, *O. sativa* and *Solanum lycopersicum* (Choi et al., 2020; Ho-Plagaro et al., 2021). Moreover, compared with the +AMF control, GA treatment significantly reduced *DLK2* expression in *L. japonicus* but further increased in *E. grandiflorum* (Figures 4A,C; Tominaga et al., 2020b). The expression of *DLK2* in GA-treated *D. carota* was slightly reduced (Figure 4B). Thus, *DLK2* expression patterns would simply mirror the GA-mediated changes in AM fungal colonization level.

### Transcriptional Responses to Exogenous Gibberellin Reflect Fungal Colonization Rates in Three Host Plant Species

To expand comparative analysis of transcriptional responses to the AM fungal colonization and GA treatment, we identified the ortholog genes with a one-to-one relationship among *L. japonicus*, *D. carota*, and *E. grandiflorum* using SonicParanoid. This resulted in 2,705 ortholog genes (Supplementary Table 2). The orthologs were designated as DEGs when they were differentially expressed at least in one condition of each host plant, resulting in 467 DEGs (Supplementary Figure 4 and Supplementary Table 5). Hierarchical clustering showed that the transcriptional responses to GA in *L. japonicus* and *D. carota* AM roots were similar to each other (Supplementary Figure 4). In GA-treated *E. grandiflorum*, the transcriptional responses were found to be close to that of +AMF samples (Supplementary Figure 4). These data are consistent with the GA-suppressed AM symbioses in *L. japonicus* and *D. carota* and GA-resistant AM symbiosis in *E. grandiflorum* (Figure 2).

Since GA highlighted the different expression patterns of the conserved and orthologous genes among the examined plants so far, we next investigated GA-mediated responses shared among them. In GA-treated plants, the shoots, petioles, and leaves were significantly elongated, as other studies showed (Supplementary Figure 5). In addition to promoting plant growth, GA-treated host plants commonly showed significantly reduced *GA20ox* expression as previous study shows (Supplementary Table 4; Cheng et al., 2015). Therefore, GA appears to have common effects on plant physiological responses in *L. japonicus*, *D. carota*, and *E. grandiflorum* as expected.

### Comparative Gene Ontology Enrichment Analysis Among the Examined Plant Species

**FIGURE 5:**
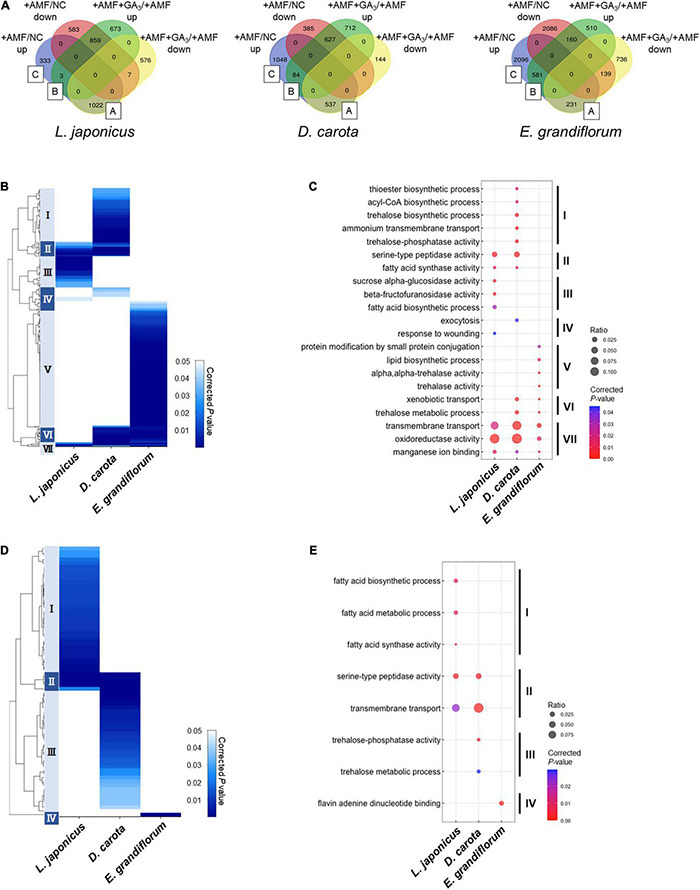
Comparisons of enriched GO among AM roots of examined host plants. **(A)** Total DEGs (| Log_2_FC| > 1, FDR < 0.01) in individual host plants during AM symbiosis at 6 wpi. Their expression patterns classify the DEGs. The values represent the number of DEGs. As for *Eustoma grandiflorum*, the values indicate the number of transcripts in *de novo* assembly data. Group A contains AM-upregulated but GA-downregulated DEGs, Group B represents AM- and GA-upregulated DEGs, and Group C indicates AM-upregulated DEGs. NC, non-colonized control; +AMF, *Rhizophagus irregularis* inoculation; +AMF +GA_3_, simultaneous application of *R. irregularis* inoculation and 1 μM GA_3_. For the determination of DEGs, transcriptomes in the host plants were compared as following: +AMF versus NC (+AMF/NC), +AMF +GA_3_ versus +AMF (+AMF +GA_3_/+AMF). **(B–E)** Hierarchical clustering of significantly enriched GO terms in the DEGs within Group A + B + C (upregulated upon AM fungal colonization) **(B)** and Group A **(D)** in each host plant at 6 wpi (corrected *p* < 0.05). The representative GO terms that enriched in each cluster of **(B,D)** were plotted in **(C,E)**, respectively. The size of circles represents ratio of DEGs enriched in a GO term to total number of DEGs. The color bar shows color-coded corrected *p*-value. The *p*-values were calculated using a two-sided hypergeometric test in the Cytoscape plugin, ClueGO, and corrected using the Benjamini–Hochberg method. For the detailed lists of DEGs and complete GO terms in each cluster, see Supplementary Table 6. AM, arbuscular mycorrhizal; DEG, differentially expressed gene; FC, fold change; FDR, false discovery rate; GA, gibberellin; GO, gene ontology.

To gain further insights into the similarity and difference in the regulation of AM symbiosis, we utilized our comparative transcriptome data to infer the physiological functions altered in the AM roots of each host plant by the GO enrichment analysis. To this end, DEGs were determined in each host plant, resulting in 4,056, 3,537, and 6,439 DEGs in *L. japonicus*, *D. carota*, and *E. grandiflorum*, respectively (Figure 5A). The relatively large number of DEGs in *E. grandiflorum* might be attributable to the redundant or alternative transcripts in *de novo* assembly (Duan et al., 2012; Ono et al., 2015), although the redundant contigs were removed from *de novo* reference data using CD-HIT (Li and Godzik, 2006; Tominaga et al., 2020b).

We classified the DEGs depending on their expression patterns (Figure 5A). Interestingly, we found that the ratio of DEGs in Group A representing AM fungus-induced but GA-suppressed genes was relatively low in *E. grandiflorum* (3.6%) compared to *L. japonicus* (25.2%) and *D. carota* (15.2%) (Figure 5A). In contrast, the percentage of DEGs in Group B representing AM fungus- and GA-induced genes was much lower in *L. japonicus* (0.074%) compared to *D. carota* (2.4%) and *E. grandiflorum* (9.0%) (Figure 5A).

As for DEGs upregulated by the AM fungal colonization (Group A + B + C), GO terms associated with membrane transport were enriched in all colonized plants (Cluster VII in Figures 5B,C and Supplementary Table 6). Some GO terms related to transport activity were still found in Group C of each host plant (Supplementary Table 6). Additionally, the analysis also detected peptidase- and fatty acid-related terms in *L. japonicus* and *D. carota* (Cluster II in Figures 5B,C). The enrichment of fatty acid-related GO term, fatty acid synthase activity, is consistent with the transcriptional promotions of *FatM* and *RAM2* in the two hosts colonized by *R. irregularis* (Figures 3A,B). On the other hand, some GO terms in Cluster II were not shared with *E. grandiflorum* (Figures 5B,C and Supplementary Table 6), which might be attributable to the fact that some homologs such as *RAM2* were not identified from the *de novo* assemble data of *E. grandiflorum* by SonicParanoid (Supplementary Figure 1A). However, some differences were found among different host plants. For example, the expressions of α-glucosidase and β-fructofuranosidase were promoted upon AM fungal colonization in *L. japonicus* (Cluster III in Figures 5B,C and Supplementary Table 6). Alternatively, *D. carota* and *E. grandiflorum* showed enhanced expressions of genes encoding trehalose biosynthetic enzymes and trehalase activity upon fungal inoculation, respectively (Cluster I, V, and VI in Figures 5B,C and Supplementary Table 6). These GO terms associating with trehalose also enriched in Group B, where genes were transcriptionally activated in both of AM fungal colonization and GA treatment (Supplementary Table 6). These results suggest that different disaccharides might be dominantly metabolized during AM symbioses: sucrose in *L. japonicus* and trehalose in the other two plants.

To compare GA-mediated change in AM-responsive genes, we next focused on Group A genes. As illustrated in the heatmap, the DEGs in the Group A of *E. grandiflorum* were significantly enriched in GO term representing flavin adenine dinucleotide binding (GO:0050660) (Figures 5D,E and Supplementary Table 6). In contrast, GO terms representing transmembrane transport and peptidase were shared in *L. japonicus* and *D. carota* (Cluster II in Figures 5D,E and Supplementary Table 6). The GO enrichment analysis again revealed that fatty acid biosynthesis was attenuated in GA-treated *L. japonicus* AM roots, corresponding to the negative effect of GA on *LjFatM* and *LjRAM2* expressions (Figures 3A, 5D,E and Supplementary Tables 4, 6). Moreover, trehalose-related genes were shown to be transcriptionally downregulated in GA-treated *D. carota* AM roots (Cluster III in Figures 5D,E and Supplementary Table 6). As for *E. grandiflorum* AM roots, trehalose metabolism was transcriptionally upregulated even in the presence of GA (Supplementary Table 6).

### Comparison of Fungal Transcriptome Obtained From Three Examined Host Plants

We found that up to 13.7% of the RNA-seq reads are mapped to the genome of *R. irregularis* (Supplementary Table 1; Maeda et al., 2018). This allowed us to compare the transcriptomes of *R. irregularis* colonizing each of GA-treated *L. japonicus*, *D. carota*, and *E. grandiflorum* against one infecting the control plants. The number of the upregulated DEGs of *R. irregularis* was relatively smaller in GA-treated *L. japonicus* compared to other plants (Supplementary Figure 6A). On the other hand, the downregulated DEGs of *R. irregularis* in GA-treated *L. japonicus* were mostly shared with *D. carota* (24.7%) compared with *E. grandiflorum* (9.9%) (Supplementary Figure 6A).

Since *R. irregularis* seemed to differentially respond to the examined plants, we next conducted the GO enrichment analysis on the fungal DEGs. Hierarchical clustering arranged by Log_2_FC revealed six clusters (Supplementary Figure 6B). We found several GO terms associated with a mitogen-activated protein kinase (MAPK) activity in Cluster IV, where upregulated DEGs in *R. irregularis* associating with GA-treated *E. grandiflorum* were dominant (Supplementary Figure 6C and Supplementary Table 7). Additionally, glycogen metabolism- and wax biosynthesis-related terms were found in the cluster. Interestingly, DEGs in Cluster VI, where numerous downregulated DEGs were found in GA-treated *L. japonicus*, were enriched in some GO terms corresponding to the elongation and oxidation of fatty acid (Supplementary Figure 6D and Supplementary Table 7). This may suggest that the allocation of host-derived fatty acids is attenuated by GA application in *L. japonicus*, which could be explained by the GA-suppressed the expression levels of genes for fatty acid biosynthesis in its AM roots (Figures 3A, 5E and Supplementary Tables 4, 6).

## Discussion

In this study, our comparative transcriptomics found a partially common transcriptional response during AM symbiosis among *L. japonicus*, *D. carota*, and *E. grandiflorum* roots in the absence of GA. A set of known AM symbiosis-related genes, *RAM1*, *RAD1*, *Vpy*, *PT4*, *AMT*s, *STR*, and *STR2*, conserved in the tested plants were transcriptionally promoted upon AM fungal colonization (Figure 3). These genes have been also shown to be transcriptionally upregulated during AM symbiosis in *L. japonicus*, *M. truncatula*, tomato, rice, and *Poncirus trifoliata* (Sugimura and Saito, 2017; An et al., 2018). Another study revealed the conservation of *RAD1*, *STR*, and *STR2* in broad AM host lineages across vascular plants and bryophytes and suggested their common functions in AM symbiosis; these AM symbiosis-related genes would comparably function in establishing AM symbiosis as well (Radhakrishnan et al., 2020). As for the function of the conserved genes, *CBX1*, *WRI5s*, *STR*, and *STR2* have been shown to be required for the full development of arbuscule by regulating fatty acid biosynthesis and transfer to AM fungi (Bravo et al., 2017; Luginbuehl et al., 2017; Jiang Y. et al., 2018; Xue et al., 2018). Additionally, the expression levels of genes encoding phosphate and ammonium transporters were enhanced by AM fungal colonization (Figure 3 and Supplementary Table 4), which would contribute to the host growth promotion regardless of the distinct AM morphotypes as shown in a previous report (Figure 1E; Hong et al., 2012). Taken together, nutrient exchange between the host plants and AM fungi would be commonly essential to establish AM symbiosis among the phylogenetically distant host plants. Especially, the capability of supplying fatty acids to AM fungi appears to be indispensable for the mutualism because AM fungi utilize lipids for their growth and reproduction (Kameoka et al., 2019b; Sugiura et al., 2020).

**FIGURE 6:**
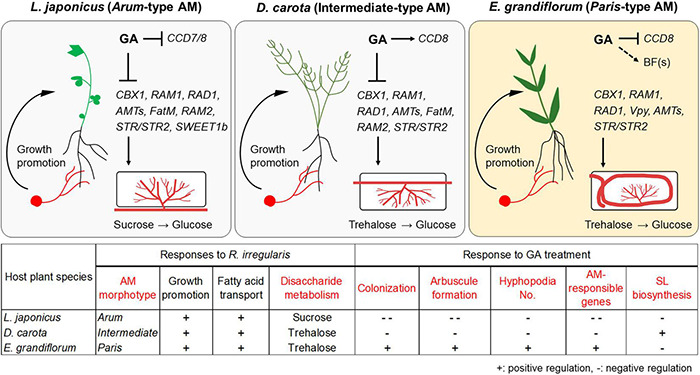
Proposed model for conserved and divergent responses to AM fungi and exogenous GA in genetically distant host plants at 6 wpi. The hypothetical model and table represent common and different responses to *Rhizophagus irregularis* colonization and exogenous GA treatment in *Lotus japonicus* (*Arum*-type), *Daucus carota* (Intermediate-type), and *Eustoma grandiflorum* (*Paris*-type) AM roots. AM fungal colonization contributed to growth promotion in each tested plant forming the distinct AM morphotypes. However, GA treatment suppressed the AM fungal colonization in *L. japonicus* and *D. carota*. In contrast, GA treatment also promoted mycorrhization in *E. grandiflorum*. These alterations in colonization levels were consistent with the expression levels of the conserved downstream genes such as *RAM1*. Interestingly, our findings indicate that the upstream regulation of the symbiosis-related genes would be resistant to GA in *E. grandiflorum*, but vulnerable to GA in the others. On the other hand, GA transcriptionally inhibited SL biosynthetic genes (*CCD7* and/or *CCD8*) in *L. japonicus* and *E. grandiflorum*, implying the existence of unidentified branching factors in *E. grandiflorum*. Moreover, disaccharides mainly metabolized during AM symbiosis might be different: sucrose and trehalose in *L. japonicus* and *D. carota*/*E. grandiflorum*. Red words in the table indicate different traits and responses found among the examined host plants in this study. +, positive regulation; –, negative regulation. The figures were created with BioRender.com. AM, arbuscular mycorrhizal; GA, gibberellin; SL, strigolactone.

Nevertheless, GA treatment negatively and positively regulated the AM fungal colonization in *L. japonicus*/*D. carota* and *E. grandiflorum*, respectively, which were consistent with the expression patterns of the conserved genes such as *RAM1* (Figures 2D, 3). Recently, CYCLOPS required for both AM symbiosis and root nodule symbiosis has been reported to bind the *cis*-element on *LjRAM1* promoter and upregulate gene expression in concert with a Ca^+2^/calmodulin-dependent protein kinase (CCaMK) and a GA-degradable repressor of GA signaling, DELLA protein (Silverstone et al., 2001; Achard and Genschik, 2009; Jin et al., 2016; Pimprikar et al., 2016). The involvement of DELLA in the complex is thought to trigger the GA-mediated inhibition of *RAM1* expression, resulting in the severe suppression of AM fungal accommodation. The binding of DELLAs to the CCaMK-CYCLOPS complex has been also demonstrated in nodule symbiosis (Jin et al., 2016). *D. carota* and *L. japonicus* showed reduced rates of AM fungal colonization and expression levels of *RAM1*, indicating that the GA-mediated transcriptional regulation of downstream genes would be common (Figure 6). However, the expression levels of *RAM1* and some of the downstream genes were significantly or slightly promoted in GA-treated *E. grandiflorum* (Figure 3C). Although this arose an idea that GA directly modulates the expression of the downstream genes, the enhanced AM fungal colonization in GA-treated *E. grandiflorum* possibly contributed to the result since the downstream genes were responsible to AM fungal colonization levels (Figures 2D, 3 and Supplementary Table 4). On the other hand, the transcriptional regulation of the downstream genes in *E. grandiflorum* would be resistant to exogenous GA since no inhibitory effect of GA on AM symbiosis was observed in the host plant except for SL production (Figures 1–4 and Supplementary Figure 3C). Therefore, DELLA might be dispensable for or inhibiting the expression of *RAM1* in *E. grandiflorum*, while this study could not uncover the DELLA function in the host plant. In fact, stabilizing DELLA proteins in *E. grandiflorum* suppresses AM fungal colonization and arbuscule formation (Tominaga et al., 2020a), whereas it did not change colonization levels or upregulate arbuscule formation (Takeda et al., 2015; Pimprikar et al., 2016). To clarify the upstream regulation of these TFs in *E. grandiflorum*, further investigation would be necessary.

Strigolactones are thought to potentiate pre-symbiotic fungal contact to the host roots because some mutants defect in SL biosynthesis and exudation showed delayed colonization and decreased the number of hyphopodia (Breuillin et al., 2010; Kretzschmar et al., 2012; Kobae et al., 2018). Additionally, SL biosynthesis and exudation are inhibited by exogenous GA (Ito et al., 2017). Indeed, we could confirm the inhibitory effects of GA on SL biosynthetic genes in *L. japonicus* and *E. grandiflorum* (Figure 4 and Supplementary Figure 3). Interestingly, the number of the hyphopodia was drastically induced in GA-treated *E. grandiflorum* roots as shown in our previous study (Figure 2E; Tominaga et al., 2020a). This study also suggests that SLs would not be involved in the GA-promoted fungal invasion due to the negative effect of GA on SL production (Figure 4C and Supplementary Figure 3C). We could not identify the unknown signal molecule(s) yet; however, the possible existence was assumed from the GO enrichment analysis on *R. irregularis*. As shown in Supplementary Figure 6, some GO terms representing the activity of MAPK kinase kinase, MAPK Kinase, and MAPK were detected in *R. irregularis* colonizing GA-treated *E. grandiflorum* (Supplementary Table 7). In plant pathogenic fungi, such as *Ustilago maydis* and *Magnaporthe oryzae*, MAPK cascade is required for the formation of appressoria and their virulence after perceiving some signal molecules derived from the host plants (Hamel et al., 2012; Li X. et al., 2017; Jiang C. et al., 2018). Although the necessity of the MAPK cascade in *R. irregularis* hyphopodia formation remains unclear, the fungus might sense some signal molecule(s) exudates from GA-treated *E. grandiflorum* roots. Except for *L. japonicus* and *E. grandiflorum*, this study indicated that SL biosynthesis in *D. carota* might be less sensitive to GA even though exogenous GA reduced the number of invading hyphopodia (Figures 2E, 3B and Supplementary Figure 3). A negative feedback regulation in SL biosynthesis might trigger the increase in *DcCCD8* expression upon exogenous GA at 6 wpi (Hayward et al., 2009; Proust et al., 2011). Alternatively, the reduction of SL exudation by GA might have occurred at an earlier time point than 6 wpi in *D. carota*.

Gibberellin is one of phytohormones that has versatile functions in abiotic stress responses. Light limitation makes plants accumulate GA, which results in the elongation of stem to gain efficient light for photosynthesis (Hisamatsu et al., 2005; Bou-Torrent et al., 2014; Colebrook et al., 2014; Li W. et al., 2017; Yang and Li, 2017). Interestingly, far-red treatment and *phyB* mutation have been reported to attenuate AM fungal colonization and SL production in *L. japonicus* and tomato colonized by *R. irregularis* (Nagata et al., 2015). Moreover, host plants forming *Paris*-type AM, such as Gentianaceae species, are often found in forest floor (Yamato and Iwasaki, 2002; Yamato, 2004). Thus, *Paris*-type AM symbiosis might enable the hosts to efficiently accommodate the symbionts in dark places. However, this idea should be further investigated because some plants are capable of suppressing shade-induced GA accumulation in petiole and hypocotyl elongation (Gommers et al., 2018; Paulišić et al., 2021). Recent study has also introduced that inorganic phosphate (Pi) inhibits AM symbiosis *via* GA signaling in Solanaceous model plants (Nouri et al., 2021). In contrast, *E. grandiflorum* might be capable of promoting AM fungal colonization in high Pi concentration, although this hypothesis needs to be explored. Taken together, some plants like *E. grandiflorum* might adapt to their surroundings by exploiting the GA-resistant AM fungal colonization and unidentified signal molecule(s). The investigations of regulatory mechanisms underlying AM symbiosis with environmental cues and/or life stages of host plants would be necessary for further understanding.

The loss of genes encoding enzymes required for polysaccharide degradation in AM fungi demands host plants on glucose (Kobayashi et al., 2018). In arbuscules containing cells of *M. truncatula*, AM fungi-responsive localization and the expression of *MtSucS1* and *MtSWEET1b* are thought to produce glucose and export it toward AM fungi between the symbiotic interface (An et al., 2019). In fact, our GO enrichment analysis showed activated sucrose hydrolysis during AM symbiosis in *L. japonicus*, which was supported by AM-induced *LjSucS1* and *LjSWEET1b*s expression (Figure 3A and Supplementary Table 4). In contrast, another disaccharide, trehalose, appeared to be broken down in *D. carota* and *E. grandiflorum* during AM symbiosis, as the increases in plant trehalase (*TRE1*) gene expressions were found in the host plants upon AM fungal colonization (Figure 5C and Supplementary Tables 3, 5). This difference might be attributable to the intracellular hyphal invasion in Intermediate- and *Paris*-type AM roots (Figures 1B–D and Supplementary Figures 1C–E). However, the increase in *TRE1* expression was also observed in *L. japonicus* AM roots at 6 wpi (Supplementary Table 4). Interestingly, it is known that most of the storage carbohydrates found in fungi are trehalose, and AM fungi can synthesize, metabolize, and accumulate trehalose in the spores and hyphae (Shachar-Hill et al., 1995; Bago et al., 1999; Pfeffer et al., 1999; Kameoka et al., 2019a). Additionally, the upregulation of *TRE1* expression has also been seen in *Arabidopsis thaliana* infected by a pathogenic fungus, *Plasmodiophora brassicae*, which is considered as the maintenance of sugar concentration and physiological homeostasis in the roots by removing fungal-derived trehalose (Brodmann et al., 2002). On the other hand, the suppression of the trehalose precursor trehalose-6-phosphate (T6P) production has been found in *L. japonicus* AM roots and predicted to be related to the decomposition of starch into glucose for AM fungi (Kolbe et al., 2005; Handa et al., 2015), indicating TRE1 would be involved in the regulation of symbiotic glucose metabolism. However, most of trehalose-6-phosphate synthases that catalyze T6P production were not transcriptionally suppressed at in *D. carota* and *E. grandiflorum* at 6 wpi (Supplementary Table 4). This suggests that the *TRE1* expression enhanced during AM symbiosis might not be involved in the starch degradation. Therefore, TRE1 might be required to reduce AM fungi-derived trehalose concentration in the host plants, for example, when arbuscules are hydrolyzed in a short period (Kobae et al., 2014; Floss et al., 2017).

In summary, a particular set of conserved AM symbiosis-related genes would commonly function to accommodate AM fungi in the phylogenetically distant AM host plants regardless of distinct AM morphotypes. However, our transcriptomics and GA treatment indicate the GA-mediated different molecular mechanisms regulating the conserved AM symbiosis-related genes between *L. japonicus*/*D. carota* and *E. grandiflorum* (Figure 6). These findings advance the comprehensive understanding of transcriptomic regulation and the diversity of GA-mediated effects on AM symbioses among host plants. Additionally, AM fungal traits sometimes affect AM morphotype formed in a single host species (Cavagnaro et al., 2001; Dickson, 2004; Smith et al., 2004; Kubota et al., 2005; Hong et al., 2012). Thus, the comparison of GA-mediated regulations underlying AM symbioses using a single host species would be expected to further support the causal relationship between AM-morphotyped and the different GA-mediated regulation of symbiosis in the next study.

## Data Availability Statement

The original contributions presented in the study are publicly available. The nucleotide sequence data obtained from our transcriptome analysis has been deposited into the DDBJ Sequence Read Archive under the accession number DRA012117. *De novo* assembly and annotation list of *E. grandiflorum* are available on Open Science Foundation with DOI:10.17605/OSF.IO/TQ7XJ or https://osf.io/tq7xj/?view_only=b6bec888fd80417ea636c3b6b58f07c1.

## Author Contributions

TT and HK conceived and designed the experiments. TT, YS, YH, and AM performed the experiments. TT, CM, KY, SS, and AM analyzed the sequencing data. TT, CM, AM, and HK wrote the manuscript. All authors approved the final manuscript.

## Conflict of Interest

The authors declare that the research was conducted in the absence of any commercial or financial relationships that could be construed as a potential conflict of interest.

## Publisher’s Note

All claims expressed in this article are solely those of the authors and do not necessarily represent those of their affiliated organizations, or those of the publisher, the editors and the reviewers. Any product that may be evaluated in this article, or claim that may be made by its manufacturer, is not guaranteed or endorsed by the publisher.

## Footnotes

1
http://imagej.nih.gov/ij

2
https://phytozome-next.jgi.doe.gov

3
https://fungi.ensembl.org/index.html

4
http://bioinformatics.psb.ugent.be/webtools/Venn

## References

[B1] Achard P. Genschik P. (2009). Releasing the brakes of plant growth: how GAs shutdown DELLA proteins. *J. Exp. Bot.* 60 1085–1092. 10.1093/jxb/ern301 19043067

[B2] Akiyama K. Matsuzaki K. Hayashi H. (2005). Plant sesquiterpenes induce hyphal branching in arbuscular mycorrhizal fungi. *Nature* 435 824–827. 10.1038/nature03608 15944706

[B3] Al-Babili S. Bouwmeester H. J. (2015). Strigolactones, a novel carotenoid-derived plant hormone. *Annu Rev Plant Biol.* 66 161–186. 10.1146/annurev-arplant-043014-114759 25621512

[B4] Alder A. Jamil M. Marzorati M. Bruno M. Vermathen M. Bigler P. (2012). The path from β-carotene to carlactone, a strigolactone-like plant hormone. *Science* 335 1348–1351. 10.1126/science.1218094 22422982

[B5] Alexa A. Rahnenfuhrer J. Lengauer T. (2006). Improved scoring of functional groups from gene expression data by decorrelating GO graph structure. *Bioinformatics* 22 1600–1607. 10.1093/bioinformatics/btl140 16606683

[B6] An J. Sun M. van Velzen R. Ji C. Zheng Z. Limpens E. (2018). Comparative transcriptome analysis of *Poncirus trifoliata* identifies a core set of genes involved in arbuscular mycorrhizal symbiosis. *J. Exp. Bot.* 69 5255–5264. 10.1093/jxb/ery283 30312435 PMC6184448

[B7] An J. Zeng T. Ji C. de Graaf S. Zheng Z. Xiao T. T. (2019). A *Medicago truncatula* SWEET transporter implicated in arbuscule maintenance during arbuscular mycorrhizal symbiosis. *New Phytol.* 224 396–408. 10.1111/nph.15975 31148173

[B8] Auldridge M. E. Block A. Vogel J. T. Dabney-Smith C. Mila I. Bouzayen M. (2006). Characterization of three members of the Arabidopsis carotenoid cleavage dioxygenase family demonstrates the divergent roles of this multifunctional enzyme family. *Plant J.* 45 982–993. 10.1111/j.1365-313X.2006.02666.x 16507088

[B9] Bago B. Pfeffer P. E. Douds D. D. Jr. Brouillette J. Bécard G. Shachar-Hill Y. (1999). Carbon metabolism in spores of the arbuscular mycorrhizal fungus *Glomus intraradices* as revealed by nuclear magnetic resonance spectroscopy. *Plant Physiol.* 121 263–272. 10.1104/pp.121.1.263 10482682 PMC59376

[B10] Baier M. C. Keck M. Godde V. Niehaus K. Kuster H. Hohnjec N. (2010). Knockdown of the symbiotic sucrose synthase MtSucS1 affects arbuscule maturation and maintenance in mycorrhizal roots of *Medicago truncatula*. *Plant Physiol.* 152 1000–1014. 10.1104/pp.109.149898 20007443 PMC2815868

[B11] Besserer A. Becard G. Jauneau A. Roux C. Sejalon-Delmas N. (2008). GR24, a synthetic analog of strigolactones, stimulates the mitosis and growth of the arbuscular mycorrhizal fungus *Gigaspora rosea* by boosting its energy metabolism. *Plant Physiol.* 148 402–413. 10.1104/pp.108.121400 18614712 PMC2528133

[B12] Bindea G. Galon J. Mlecnik B. (2013). CluePedia Cytoscape plugin: pathway insights using integrated experimental and in silico data. *Bioinformatics* 29 661–663. 10.1093/bioinformatics/btt019 23325622 PMC3582273

[B13] Bindea G. Mlecnik B. Hackl H. Charoentong P. Tosolini M. Kirilovsky A. (2009). ClueGO: a Cytoscape plug-in to decipher functionally grouped gene ontology and pathway annotation networks. *Bioinformatics* 25 1091–1093. 10.1093/bioinformatics/btp101 19237447 PMC2666812

[B14] Booker J. Auldridge M. Wills S. McCarty D. Klee H. Leyser O. (2004). MAX3/CCD7 is a carotenoid cleavage dioxygenase required for the synthesis of a novel plant signaling molecule. *Curr. Biol.* 14 1232–1238. 10.1016/j.cub.2004.06.061 15268852

[B15] Booker J. Sieberer T. Wright W. Williamson L. Willett B. Stirnberg P. (2005). *MAX1* encodes a cytochrome P450 family member that acts downstream of *MAX3*/*4* to produce a carotenoid-derived branch-inhibiting hormone. *Dev. Cell.* 8 443–449. 10.1016/j.devcel.2005.01.009 15737939

[B16] Bou-Torrent J. Galstyan A. Gallemí M. Cifuentes-Esquivel N. Molina-Contreras M. J. Salla-Martret M. (2014). Plant proximity perception dynamically modulates hormone levels and sensitivity in *Arabidopsis*. *J. Exp. Bot.* 65 2937–2947. 10.1093/jxb/eru083 24609653 PMC4056540

[B17] Bravo A. Brands M. Wewer V. Dormann P. Harrison M. J. (2017). Arbuscular mycorrhiza-specific enzymes FatM and RAM2 fine-tune lipid biosynthesis to promote development of arbuscular mycorrhiza. *New Phytol.* 214 1631–1645. 10.1111/nph.14533 28380681

[B18] Breuillin F. Schramm J. Hajirezaei M. Ahkami A. Favre P. Druege U. (2010). Phosphate systemically inhibits development of arbuscular mycorrhiza in *Petunia hybrida* and represses genes involved in mycorrhizal functioning. *Plant J.* 64 1002–1017. 10.1111/j.1365-313X.2010.04385.x 21143680

[B19] Brodmann A. Schuller A. Ludwig-Müller J. Aeschbacher R. A. Wiemken A. Boller T. (2002). Induction of trehalase in *Arabidopsis* plants infected with the trehalose-producing pathogen *Plasmodiophora brassicae*. *Mol. Plant Microbe Interact.* 15 693–700. 10.1094/mpmi.2002.15.7.693 12118885

[B20] Brundrett M. C. Tedersoo L. (2018). Evolutionary history of mycorrhizal symbioses and global host plant diversity. *New Phytol.* 220 1108–1115. 10.1111/nph.14976 29355963

[B21] Cavagnaro T. R. Gao L. L. Smith F. A. Smith S. E. (2001). Morphology of arbuscular mycorrhizas is influenced by fungal identity. *New Phytologist* 151 469–475. 10.1046/j.0028-646x.2001.00191.x

[B22] Chen S. Zhou Y. Chen Y. Gu J. (2018). fastp: an ultra-fast all-in-one FASTQ preprocessor. *Bioinformatics* 34 i884–i890. 10.1093/bioinformatics/bty560 30423086 PMC6129281

[B23] Cheng C. Jiao C. Singer S. D. Gao M. Xu X. Zhou Y. (2015). Gibberellin-induced changes in the transcriptome of grapevine (Vitis labrusca x V. vinifera) cv. Kyoho flowers. *BMC Genomics* 16:128. 10.1186/s12864-015-1324-8 25888129 PMC4348105

[B24] Choi J. Lee T. Cho J. Servante E. K. Pucker B. Summers W. (2020). The negative regulator SMAX1 controls mycorrhizal symbiosis and strigolactone biosynthesis in rice. *Nat. Commun.* 11:2114. 10.1038/s41467-020-16021-1 32355217 PMC7193599

[B25] Colebrook E. H. Thomas S. G. Phillips A. L. Hedden P. (2014). The role of gibberellin signalling in plant responses to abiotic stress. *J. Exp. Biol.* 217 67–75. 10.1242/jeb.089938 24353205

[B26] Cosentino S. Iwasaki W. (2019). SonicParanoid: fast, accurate and easy orthology inference. *Bioinformatics* 35 149–151. 10.1093/bioinformatics/bty631 30032301 PMC6298048

[B27] Dickson S. (2004). The *Arum*-*Paris* continuum of mycorrhizal symbioses. *New Phytologist.* 163 187–200. 10.1111/j.1469-8137.2004.01095.x 33873792

[B28] Dickson S. Smith F. A. Smith S. E. (2007). Structural differences in arbuscular mycorrhizal symbioses: more than 100 years after Gallaud, where next? *Mycorrhiza* 17 375–393. 10.1007/s00572-007-0130-9 17476535

[B29] Dobin A. Davis C. A. Schlesinger F. Drenkow J. Zaleski C. Jha S. (2013). STAR: ultrafast universal RNA-seq aligner. *Bioinformatics* 29 15–21. 10.1093/bioinformatics/bts635 23104886 PMC3530905

[B30] Duan J. Xia C. Zhao G. Jia J. Kong X. (2012). Optimizing *de novo* common wheat transcriptome assembly using short-read RNA-Seq data. *BMC Genomics* 13:392. 10.1186/1471-2164-13-392 22891638 PMC3485621

[B31] Ezawa T. Saito K. (2018). How do arbuscular mycorrhizal fungi handle phosphate? New insight into fine-tuning of phosphate metabolism. *New Phytol.* 220 1116–1121. 10.1111/nph.15187 29701874

[B32] Floková K. Shimels M. Andreo Jimenez B. Bardaro N. Strnad M. Novák O. (2020). An improved strategy to analyse strigolactones in complex sample matrices using UHPLC-MS/MS. *Plant Methods* 16:125. 10.1186/s13007-020-00669-3 32963580 PMC7499983

[B33] Floss D. S. Gomez S. K. Park H. J. MacLean A. M. Muller L. M. Bhattarai K. K. (2017). A Transcriptional Program for Arbuscule Degeneration during AM Symbiosis Is Regulated by MYB1. *Curr. Biol.* 27 1206–1212. 10.1016/j.cub.2017.03.003 28392110

[B34] Floss D. S. Levy J. G. Levesque-Tremblay V. Pumplin N. Harrison M. J. (2013). DELLA proteins regulate arbuscule formation in arbuscular mycorrhizal symbiosis. *Proc. Natl. Acad. Sci. U S A.* 110 E5025–E5034. 10.1073/pnas.1308973110 24297892 PMC3870710

[B35] Galili T. O’Callaghan A. Sidi J. Sievert C. (2018). heatmaply: an R package for creating interactive cluster heatmaps for online publishing. *Bioinformatics* 34 1600–1602. 10.1093/bioinformatics/btx657 29069305 PMC5925766

[B36] Gobbato E. Wang E. Higgins G. Bano S. A. Henry C. Schultze M. (2013). *RAM1* and *RAM2* function and expression during arbuscular mycorrhizal symbiosis and *Aphanomyces euteiches* colonization. *Plant Signal. Behav.* 8:26049. 10.4161/psb.26049 24270627 PMC4091073

[B37] Gommers C. M. M. Buti S. Tarkowská D. Pěnčík A. Banda J. P. Arricastres V. (2018). Organ-specific phytohormone synthesis in two *Geranium* species with antithetical responses to far-red light enrichment. *Plant Direct.* 2:e00066. 10.1002/pld3.66 31245741 PMC6508794

[B38] Grabherr M. G. Haas B. J. Yassour M. Levin J. Z. Thompson D. A. Amit I. (2011). Full-length transcriptome assembly from RNA-Seq data without a reference genome. *Nat. Biotechnol.* 29 644–652. 10.1038/nbt.1883 21572440 PMC3571712

[B39] Haas B. J. Papanicolaou A. Yassour M. Grabherr M. Blood P. D. Bowden J. (2013). *De novo* transcript sequence reconstruction from RNA-seq using the Trinity platform for reference generation and analysis. *Nat. Protoc.* 8 1494–1512. 10.1038/nprot.2013.084 23845962 PMC3875132

[B40] Halouzka R. Zeljkovic S. C. Klejdus B. Tarkowski P. (2020). Analytical methods in strigolactone research. *Plant Methods* 16:76. 10.1186/s13007-020-00616-2 32514284 PMC7257151

[B41] Hamel L. P. Nicole M. C. Duplessis S. Ellis B. E. (2012). Mitogen-activated protein kinase signaling in plant-interacting fungi: distinct messages from conserved messengers. *Plant Cell* 24 1327–1351. 10.1105/tpc.112.096156 22517321 PMC3398478

[B42] Handa Y. Nishide H. Takeda N. Suzuki Y. Kawaguchi M. Saito K. (2015). RNA-seq Transcriptional Profiling of an Arbuscular Mycorrhiza Provides Insights into Regulated and Coordinated Gene Expression in Lotus japonicus and Rhizophagus irregularis. *Plant Cell Physiol.* 56 1490–1511. 26009592 10.1093/pcp/pcv071

[B43] Hart A. J. Ginzburg S. Xu M. S. Fisher C. R. Rahmatpour N. Mitton J. B. (2020). EnTAP: Bringing faster and smarter functional annotation to non-model eukaryotic transcriptomes. *Mol. Ecol analyse strigolactones in c.Resour.* 20 591–604. 10.1111/1755-0998.13106 31628884

[B44] Hayward A. Stirnberg P. Beveridge C. Leyser O. (2009). Interactions between auxin and strigolactone in shoot branching control. *Plant Physiol.* 151 400–412. 10.1104/pp.109.137646 19641034 PMC2735998

[B45] Hisamatsu T. King R. W. Helliwell C. A. Koshioka M. (2005). The involvement of gibberellin 20-oxidase genes in phytochrome-regulated petiole elongation of Arabidopsis. *Plant Physiol.* 138 1106–1116. 10.1104/pp.104.059055 15923331 PMC1150424

[B46] Hohnjec N. Perlick A. M. Puhler A. Kuster H. (2003). The *Medicago truncatula* sucrose synthase gene *MtSucS1* is activated both in the infected region of root nodules and in the cortex of roots colonized by arbuscular mycorrhizal fungi. *Mol. Plant Microbe Interact.* 16 903–915. 10.1094/MPMI.2003.16.10.903 14558692

[B47] Hong J. J. Park Y. S. Bravo A. Bhattarai K. K. Daniels D. A. Harrison M. J. (2012). Diversity of morphology and function in arbuscular mycorrhizal symbioses in *Brachypodium distachyon*. *Planta* 236 851–865. 10.1007/s00425-012-1677-z 22711284

[B48] Ho-Plagaro T. Morcillo R. J. L. Tamayo-Navarrete M. I. Huertas R. Molinero-Rosales N. Lopez-Raez J. A. (2021). DLK2 regulates arbuscule hyphal branching during arbuscular mycorrhizal symbiosis. *New Phytol.* 229 548–562. 10.1111/nph.16938 32966595

[B49] Iorizzo M. Ellison S. Senalik D. Zeng P. Satapoomin P. Huang J. (2016). A high-quality carrot genome assembly provides new insights into carotenoid accumulation and asterid genome evolution. *Nat. Genet.* 48 657–666. 10.1038/ng.3565 27158781

[B50] Ito S. Yamagami D. Umehara M. Hanada A. Yoshida S. Sasaki Y. (2017). Regulation of Strigolactone Biosynthesis by Gibberellin Signaling. *Plant Physiol.* 174 1250–1259. 10.1104/pp.17.00301 28404726 PMC5462043

[B51] Jiang C. Zhang X. Liu H. Xu J. R. (2018). Mitogen-activated protein kinase signaling in plant pathogenic fungi. *PLoS Pathog.* 14:e1006875. 10.1371/journal.ppat.1006875 29543901 PMC5854419

[B52] Jiang Y. Xie Q. Wang W. Yang J. Zhang X. Yu N. (2018). *Medicago* AP2-domain transcription factor WRI5a is a master regulator of lipid biosynthesis and transfer during mycorrhizal symbiosis. *Mol. Plant.* 11 1344–1359. 10.1016/j.molp.2018.09.006 30292683

[B53] Jin Y. Liu H. Luo D. Yu N. Dong W. Wang C. (2016). DELLA proteins are common components of symbiotic rhizobial and mycorrhizal signalling pathways. *Nat. Commun.* 7:12433. 10.1038/ncomms12433 27514472 PMC4990646

[B54] Kameoka H. Kyozuka J. (2015). Downregulation of rice *DWARF 14 LIKE* suppress mesocotyl elongation via a strigolactone independent pathway in the dark. *J Genet Genomics* 42 119–124. 10.1016/j.jgg.2014.12.003 25819088

[B55] Kameoka H. Tsutsui I. Saito K. Kikuchi Y. Handa Y. Ezawa T. (2019b). Stimulation of asymbiotic sporulation in arbuscular mycorrhizal fungi by fatty acids. *Nat. Microbiol.* 4 1654–1660. 10.1038/s41564-019-0485-7 31235957

[B56] Kameoka H. Maeda T. Okuma N. Kawaguchi M. (2019a). Structure-specific regulation of nutrient transport and metabolism in arbuscular mycorrhizal fungi. *Plant Cell Physiol.* 60 2272–2281. 10.1093/pcp/pcz122 31241164

[B57] Kobae Y. Gutjahr C. Paszkowski U. Kojima T. Fujiwara T. Hata S. (2014). Lipid droplets of arbuscular mycorrhizal fungi emerge in concert with arbuscule collapse. *Plant Cell Physiol.* 55 1945–1953. 10.1093/pcp/pcu123 25231957

[B58] Kobae Y. Hata S. (2010). Dynamics of periarbuscular membranes visualized with a fluorescent phosphate transporter in arbuscular mycorrhizal roots of rice. *Plant Cell Physiol.* 51 341–353. 10.1093/pcp/pcq013 20097910

[B59] Kobae Y. Kameoka H. Sugimura Y. Saito K. Ohtomo R. Fujiwara T. (2018). Strigolactone biosynthesis genes of rice are required for the punctual entry of arbuscular mycorrhizal fungi into the roots. *Plant Cell Physiol.* 59 544–553. 10.1093/pcp/pcy001 29325120

[B60] Kobayashi Y. Maeda T. Yamaguchi K. Kameoka H. Tanaka S. Ezawa T. (2018). The genome of *Rhizophagus clarus* HR1 reveals a common genetic basis for auxotrophy among arbuscular mycorrhizal fungi. *BMC Genomics* 19:465. 10.1186/s12864-018-4853-0 29914365 PMC6007072

[B61] Kolbe A. Tiessen A. Schluepmann H. Paul M. Ulrich S. Geigenberger P. (2005). Trehalose 6-phosphate regulates starch synthesis via posttranslational redox activation of ADP-glucose pyrophosphorylase. *Proc. Natl. Acad. Sci. U S A.* 102 11118–11123. 10.1073/pnas.0503410102 16046541 PMC1180623

[B62] Kretzschmar T. Kohlen W. Sasse J. Borghi L. Schlegel M. Bachelier J. B. (2012). A petunia ABC protein controls strigolactone-dependent symbiotic signalling and branching. *Nature* 483 341–344. 10.1038/nature10873 22398443

[B63] Kubota M. McGonigle T. P. Hyakumachi M. (2005). Co-occurrence of Arum- and Paris-type morphologies of arbuscular mycorrhizae in cucumber and tomato. *Mycorrhiza* 15 73–77. 10.1007/s00572-004-0299-0 15007710

[B64] Kurihara D. Mizuta Y. Sato Y. Higashiyama T. (2015). ClearSee: a rapid optical clearing reagent for whole-plant fluorescence imaging. *Development* 142 4168–4179. 10.1242/dev.127613 26493404 PMC4712841

[B65] Langmead B. Salzberg S. L. (2012). Fast gapped-read alignment with Bowtie 2. *Nat. Methods* 9 357–359. 10.1038/nmeth.1923 22388286 PMC3322381

[B66] Li B. Ruotti V. Stewart R. M. Thomson J. A. Dewey C. N. (2010). RNA-Seq gene expression estimation with read mapping uncertainty. *Bioinformatics* 26 493–500. 10.1093/bioinformatics/btp692 20022975 PMC2820677

[B67] Li H. Jiang F. Wu P. Wang K. Cao Y. (2020). A high-quality genome sequence of model Legume *Lotus japonicus* (MG-20) provides insights into the evolution of root nodule Ssmbiosis. *Genes* 11:11050483. 10.3390/genes11050483 32365501 PMC7290416

[B68] Li H. T. Yi T. S. Gao L. M. Ma P. F. Zhang T. Yang J. B. (2019). Origin of angiosperms and the puzzle of the Jurassic gap. *Nat. Plants* 5 461–470. 10.1038/s41477-019-0421-0 31061536

[B69] Li W. Godzik A. (2006). Cd-hit: a fast program for clustering and comparing large sets of protein or nucleotide sequences. *Bioinformatics* 22 1658–1659. 10.1093/bioinformatics/btl158 16731699

[B70] Li W. Katin-Grazzini L. Gu X. Wang X. El-Tanbouly R. Yer H. (2017). Transcriptome analysis reveals differential gene expression and a possible role of gibberellins in a shade-tolerant mutant of perennial ryegrass. *Front. Plant Sci.* 8:868. 10.3389/fpls.2017.00868 28603533 PMC5445233

[B71] Li X. Gao C. Li L. Liu M. Yin Z. Zhang H. (2017). MoEnd3 regulates appressorium formation and virulence through mediating endocytosis in rice blast fungus *Magnaporthe oryzae*. *PLoS Pathog.* 13:e1006449. 10.1371/journal.ppat.1006449 28628655 PMC5491321

[B72] Liao Y. Smyth G. K. Shi W. (2014). featureCounts: an efficient general purpose program for assigning sequence reads to genomic features. *Bioinformatics* 30 923–930. 10.1093/bioinformatics/btt656 24227677

[B73] Luginbuehl L. H. Menard G. N. Kurup S. Van Erp H. Radhakrishnan G. V. Breakspear A. (2017). Fatty acids in arbuscular mycorrhizal fungi are synthesized by the host plant. *Science* 356 1175–1178. 28596311 10.1126/science.aan0081

[B74] Luginbuehl L. H. Oldroyd G. E. D. (2017). Understanding the arbuscule at the heart of endomycorrhizal symbioses in plants. *Curr. Biol.* 27 R952–R963. 28898668 10.1016/j.cub.2017.06.042

[B75] Maeda T. Kobayashi Y. Kameoka H. Okuma N. Takeda N. Yamaguchi K. (2018). Evidence of non-tandemly repeated rDNAs and their intragenomic heterogeneity in *Rhizophagus irregularis*. *Commun. Biol.* 1:87. 10.1038/s42003-018-0094-7 PMC6123716 30271968

[B76] Mcgonigle T. P. Miller M. H. Evans D. G. Fairchild G. L. Swan J. A. (1990). A new method which gives an objective-measure of colonization of roots by vesicular arbuscular mycorrhizal fungi. *New Phytologist* 115 495–501. 10.1111/j.1469-8137.1990.tb00476.x 33874272

[B77] Muller L. M. Campos-Soriano L. Levesque-Tremblay V. Bravo A. Daniels D. A. Pathak S. (2020). Constitutive overexpression of *RAM1* leads to an increase in arbuscule density in *Brachypodium distachyon*. *Plant Physiol.* 184 1263–1272. 10.1104/pp.20.00997 32873628 PMC7608154

[B78] Nagata M. Yamamoto N. Shigeyama T. Terasawa Y. Anai T. Sakai T. (2015). Red/far red light controls arbuscular mycorrhizal colonization via jasmonic acid and strigolactone signaling. *Plant Cell Physiol.* 56 2100–2109. 10.1093/pcp/pcv135 26412782

[B79] Nouri E. Surve R. Bapaume L. Stumpe M. Chen M. Zhang Y. (2021). Phosphate suppression of arbuscular mycorrhizal symbiosis involves gibberellic acid signalling. *Plant Cell Physiol.* 2021:63. 10.1093/pcp/pcab063 34037236 PMC8504448

[B80] Ono H. Ishii K. Kozaki T. Ogiwara I. Kanekatsu M. Yamada T. (2015). Removal of redundant contigs from de novo RNA-Seq assemblies via homology search improves accurate detection of differentially expressed genes. *BMC Genomics* 16:1031. 10.1186/s12864-015-2247-0 26637306 PMC4670531

[B81] Park H. J. Floss D. S. Levesque-Tremblay V. Bravo A. Harrison M. J. (2015). Hyphal branching during arbuscule development requires *Reduced arbuscular mycorrhiza1*. *Plant Physiol.* 169 2774–2788. 26511916 10.1104/pp.15.01155 PMC4677905

[B82] Paulišić S. Qin W. Arora Verasztó H. Then C. Alary B. Nogue F. (2021). Adjustment of the PIF7-HFR1 transcriptional module activity controls plant shade adaptation. *Embo* J. 40:e104273. 10.15252/embj.2019104273 33264441 PMC7780144

[B83] Pfeffer P. E. Douds D. D. Jr. Becard G. Shachar-Hill Y. (1999). Carbon uptake and the metabolism and transport of lipids in an arbuscular mycorrhiza. *Plant Physiol.* 120 587–598. 10.1104/pp.120.2.587 10364411 PMC59298

[B84] Pimprikar P. Carbonnel S. Paries M. Katzer K. Klingl V. Bohmer M. J. (2016). A CCaMK-CYCLOPS-DELLA complex activates transcription of *RAM1* to regulate arbuscule branching. *Curr. Biol.* 26 987–998. 10.1016/j.cub.2016.01.069 27020747

[B85] Proust H. Hoffmann B. Xie X. Yoneyama K. Schaefer D. G. Yoneyama K. (2011). Strigolactones regulate protonema branching and act as a quorum sensing-like signal in the moss *Physcomitrella patens*. *Development* 138 1531–1539. 10.1242/dev.058495 21367820

[B86] Radhakrishnan G. V. Keller J. Rich M. K. Vernie T. Mbadinga Mbadinga D. L. Vigneron N. (2020). An ancestral signalling pathway is conserved in intracellular symbioses-forming plant lineages. *Nat. Plants* 6 280–289. 10.1038/s41477-020-0613-7 32123350

[B87] Rich M. K. Courty P. E. Roux C. Reinhardt D. (2017). Role of the GRAS transcription factor ATA/RAM1 in the transcriptional reprogramming of arbuscular mycorrhiza in *Petunia hybrida*. *BMC Genomics* 18:589. 10.1186/s12864-017-3988-8 28789611 PMC5549340

[B88] Rich M. K. Schorderet M. Bapaume L. Falquet L. Morel P. Vandenbussche M. (2015). The Petunia GRAS Transcription Factor ATA/RAM1 Regulates Symbiotic Gene Expression and Fungal Morphogenesis in Arbuscular Mycorrhiza. *Plant Physiol.* 168 788–797. 10.1104/pp.15.00310 25971550 PMC4741351

[B89] Roberts A. Pachter L. (2013). Streaming fragment assignment for real-time analysis of sequencing experiments. *Nat. Methods* 10 71–73. 23160280 10.1038/nmeth.2251 PMC3880119

[B90] Robinson M. D. McCarthy D. J. Smyth G. K. (2010). edgeR: a Bioconductor package for differential expression analysis of digital gene expression data. *Bioinformatics* 26 139–140. 10.1093/bioinformatics/btp616 19910308 PMC2796818

[B91] Sato D. Awad A. A. Chae S. H. Yokota T. Sugimoto Y. Takeuchi Y. (2003). Analysis of strigolactones, germination stimulants for striga and orobanche, by high-performance liquid chromatography/tandem mass spectrometry. *J. Agric. Food Chem.* 51 1162–1168. 10.1021/jf025997z 12590450

[B92] Shachar-Hill Y. Pfeffer P. E. Douds D. Osman S. F. Doner L. W. Ratcliffe R. G. (1995). Partitioning of intermediary carbon metabolism in vesicular-arbuscular mycorrhizal leek. *Plant Physiol.* 108 7–15. 12228450 10.1104/pp.108.1.7 PMC157300

[B93] Shi J. Zhao B. Zheng S. Zhang X. Wang X. Dong W. (2021). A phosphate starvation response-centered network regulates mycorrhizal symbiosis. *Cell* 2021:30. 10.1016/j.cell.2021.09.030 34644527

[B94] Silverstone A. L. Jung H. S. Dill A. Kawaide H. Kamiya Y. Sun T. P. (2001). Repressing a repressor: gibberellin-induced rapid reduction of the RGA protein in Arabidopsis. *Plant Cell* 13 1555–1566. 10.1105/tpc.010047 11449051 PMC139546

[B95] Smith F. A. Smith S. E. (1997). Tansley Review No. 96. Structural diversity in (vesicular)-arbuscular mycorrhizal symbioses. *New Phytologist* 137 373–388. 10.1046/j.1469-8137.1997.00848.x 33863081

[B96] Smith S. E. Smith F. A. Jakobsen I. (2004). Functional diversity in arbuscular mycorrhizal (AM) symbioses: the contribution of the mycorrhizal P uptake pathway is not correlated with mycorrhizal responses in growth or total P uptake. *New Phytologist* 162 511–524. 10.1111/j.1469-8137.2004.01039.x

[B97] Sugimura Y. Saito K. (2017). Comparative transcriptome analysis between *Solanum lycopersicum* L. and *Lotus japonicus* L. during arbuscular mycorrhizal development. *Soil Sci. Plant Nutrit.* 63 127–136.

[B98] Sugiura Y. Akiyama R. Tanaka S. Yano K. Kameoka H. Marui S. (2020). Myristate can be used as a carbon and energy source for the asymbiotic growth of arbuscular mycorrhizal fungi. *Proc. Natl. Acad. Sci. U S A.* 117 25779–25788. 32999061 10.1073/pnas.2006948117 PMC7568319

[B99] Takeda N. Handa Y. Tsuzuki S. Kojima M. Sakakibara H. Kawaguchi M. (2015). Gibberellins interfere with symbiosis signaling and gene expression and alter colonization by arbuscular mycorrhizal fungi in *Lotus japonicus*. *Plant Physiol.* 167 545–557. 10.1104/pp.114.247700 25527715 PMC4326748

[B100] Tominaga T. Miura C. Takeda N. Kanno Y. Takemura Y. Seo M. (2020a). Gibberellin promotes fungal entry and colonization during *Paris*-type arbuscular mycorrhizal symbiosis in *Eustoma grandiflorum*. *Plant Cell Physiol.* 61 565–575. 10.1093/pcp/pcz222 31790118

[B101] Tominaga T. Yamaguchi K. Shigenobu S. Yamato M. Kaminaka H. (2020b). The effects of gibberellin on the expression of symbiosis-related genes in *Paris*-type arbuscular mycorrhizal symbiosis in *Eustoma grandiflorum*. *Plant Signal. Behav.* 15:1784544. 10.1080/15592324.2020.1784544 32594890 PMC8550185

[B102] Trabelsi I. Yoneyama K. Abbes Z. Amri M. Xie X. Kisugi T. (2017). Characterization of strigolactones produced by *Orobanche foetida* and *Orobanche crenata* resistant faba bean (*Vicia faba* L.) genotypes and effects of phosphorous, nitrogen, and potassium deficiencies on strigolactone production. *South Afr. J. Bot.* 108 15–22. 10.1016/j.sajb.2016.09.009

[B103] Tsuzuki S. Handa Y. Takeda N. Kawaguchi M. (2016). Strigolactone-induced putative secreted protein 1 Is required for the establishment of symbiosis by the arbuscular mycorrhizal fungus *Rhizophagus irregularis*. *Mol. Plant Microbe Interact.* 29 277–286. 10.1094/MPMI-10-15-0234-R 26757243

[B104] Ueno K. Furumoto T. Umeda S. Mizutani M. Takikawa H. Batchvarova R. (2014). Heliolactone, a non-sesquiterpene lactone germination stimulant for root parasitic weeds from sunflower. *Phytochemistry* 108 122–128. 25446236 10.1016/j.phytochem.2014.09.018

[B105] Vegh A. Incze N. Fabian A. Huo H. Bradford K. J. Balazs E. (2017). Comprehensive analysis of DWARF14-LIKE2 (DLK2) reveals its functional divergence from strigolactone-related paralogs. *Front. Plant Sci.* 8:1641. 10.3389/fpls.2017.01641 28970845 PMC5609103

[B106] Wagner G. P. Kin K. Lynch V. J. (2012). Measurement of mRNA abundance using RNA-seq data: RPKM measure is inconsistent among samples. *Theory Biosci.* 131 281–285. 10.1007/s12064-012-0162-3 22872506

[B107] Wang S. Chen A. Xie K. Yang X. Luo Z. Chen J. (2020). Functional analysis of the OsNPF4.5 nitrate transporter reveals a conserved mycorrhizal pathway of nitrogen acquisition in plants. *Proc. Natl. Acad. Sci. U S A.* 117 16649–16659. 10.1073/pnas.2000926117 32586957 PMC7368293

[B108] Waters M. T. Brewer P. B. Bussell J. D. Smith S. M. Beveridge C. A. (2012a). The Arabidopsis ortholog of rice DWARF27 acts upstream of MAX1 in the control of plant development by strigolactones. *Plant Physiol.* 159 1073–1085. 10.1104/pp.112.196253 22623516 PMC3387695

[B109] Waters M. T. Nelson D. C. Scaffidi A. Flematti G. R. Sun Y. K. Dixon K. W. (2012b). Specialisation within the DWARF14 protein family confers distinct responses to karrikins and strigolactones in *Arabidopsis*. *Development* 139 1285–1295. 10.1242/dev.074567 22357928

[B110] Xue L. Klinnawee L. Zhou Y. Saridis G. Vijayakumar V. Brands M. (2018). AP2 transcription factor CBX1 with a specific function in symbiotic exchange of nutrients in mycorrhizal *Lotus japonicus*. *Proc. Natl. Acad. Sci. U S A.* 115 E9239–E9246. 10.1073/pnas.1812275115 30209216 PMC6166803

[B111] Yamato M. (2004). Morphological types of arbuscular mycorrhizal fungi in roots of weeds on vacant land. *Mycorrhiza* 14 127–131. 12774218 10.1007/s00572-003-0246-5

[B112] Yamato M. Iwasaki M. (2002). Morphological types of arbuscular mycorrhizal fungi in roots of understory plants in Japanese deciduous broadleaved forests. *Mycorrhiza* 12 291–296. 10.1007/s00572-002-0187-4 12466916

[B113] Yang C. Li L. (2017). Hormonal regulation in shade avoidance. *Front. Plant Sci.* 8:1527. 10.3389/fpls.2017.01527 28928761 PMC5591575

[B114] Yu N. Luo D. Zhang X. Liu J. Wang W. Jin Y. (2014). A DELLA protein complex controls the arbuscular mycorrhizal symbiosis in plants. *Cell Res.* 24 130–133. 10.1038/cr.2013.167 24343576 PMC3879709

