# Supplementary material for: Conservation and Diversity in Gibberellin-Mediated Transcriptional Responses Among Host Plants Forming Distinct Arbuscular Mycorrhizal Morphotypes
Source: Front Plant Sci. 2021 Dec 16;12:795695. doi: 10.3389/fpls.2021.795695 (PMC8718060; doi:10.3389/fpls.2021.795695)
Supplement: Supplementary file 8 [file Presentation_1.PDF]

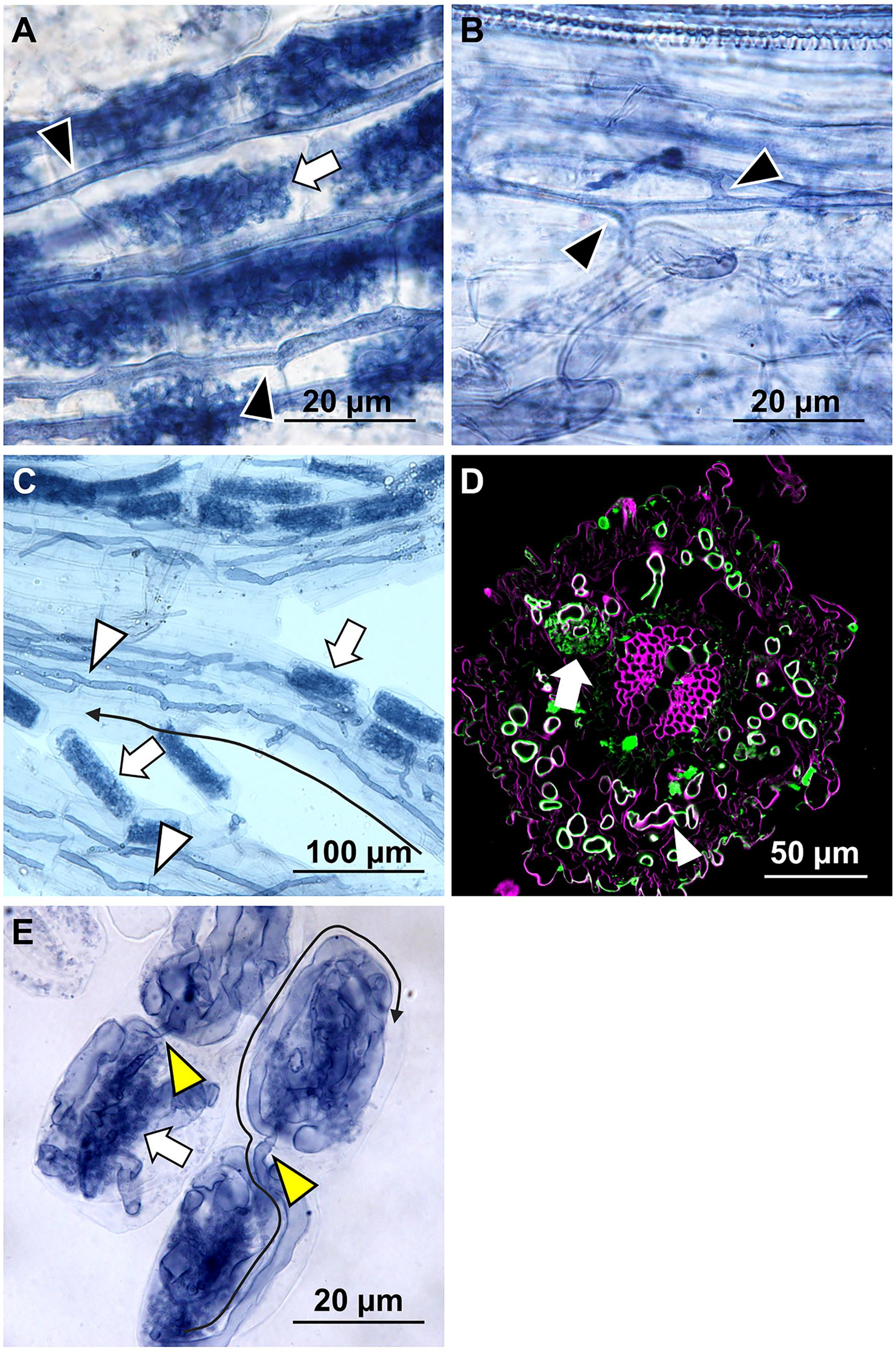

Supplementary Figure 1 | Observation of hyphal structures in root cortex of examined host plants. The AM morphotypes of each host plant colonized by *R. irregularis* were microscopically observed at 6 wpi. **(A, B)** The arbuscules and intercellular hyphae found in 0.01% ethanol-treated **(A)** and 1 µM GA_3-treated *L. japonicus* **(B)**. **(C, D)** The AM fungal morphology formed in *D. carota* roots visualized by trypan blue **(C)**, or the cross section of *D. carota* AM root stained with WGA-Alexa Fluor 488 for fungal hyphae (green) and Calcofluor White for plant cell wall (magenta) **(D)**. **(E)** Trypan blue staining of *E. grandiflorum* cortical cells infected by *R. irregularis*. Arrows, arbuscules; black arrowheads, intercellular hyphae; white arrowheads, linear and intracellular hyphae invading the cortical cells of *D. carota*; yellow arrowheads, hyphal coils directly penetrating the adjacent cortical cells of *E. grandiflorum*. Black arrows in **(C, E)** indicate the direction of intracellular hyphal elongation in *D. carota* **(C)** and *E. grandiflorum* **(E)** roots.
