# Supplementary material for: Conservation and Diversity in Gibberellin-Mediated Transcriptional Responses Among Host Plants Forming Distinct Arbuscular Mycorrhizal Morphotypes
Source: Front Plant Sci. 2021 Dec 16;12:795695. doi: 10.3389/fpls.2021.795695 (PMC8718060; doi:10.3389/fpls.2021.795695)
Supplement: Supplementary file 9 [file Presentation_2.PDF]

A

	AP2-domein TFs				GRAS TFs		Arbuscle formation/nutrient exchange									
	CBX1	*WRI5a*	*WRI5b*	*WRI5c*	*RAM1*	*RAD1*	*Vpy*	*PT4*	*AMT 2;2*	*AMT 2;3*	*FatM*	*RAM2*	*STR*	*STR2*	*SucS1*	*SWEET1b*
L. japonicus	1	1	1	1	1	1	2	1	1	1	1	1	1	1	2	3
D. carota	1		1		1	2	1	1	1	2	3	2	2	1	3	4
E. grandiflorum	1				1	2	2			1	2		2	1	2	2

B

	SL biosynthesis/transport					SL/KAR/KL signaling		
	D27	*CCD7*	*CCD8*	*MAX1*	*PDR1*	*D14*	*DLK2*	*KAI2*
L. japonicus	1	1	1	2	2	1	1	2
D. carota	2	1	1	1	1	1	1	1
E. grandiflorum	2	2	2	1	1	2	4	1

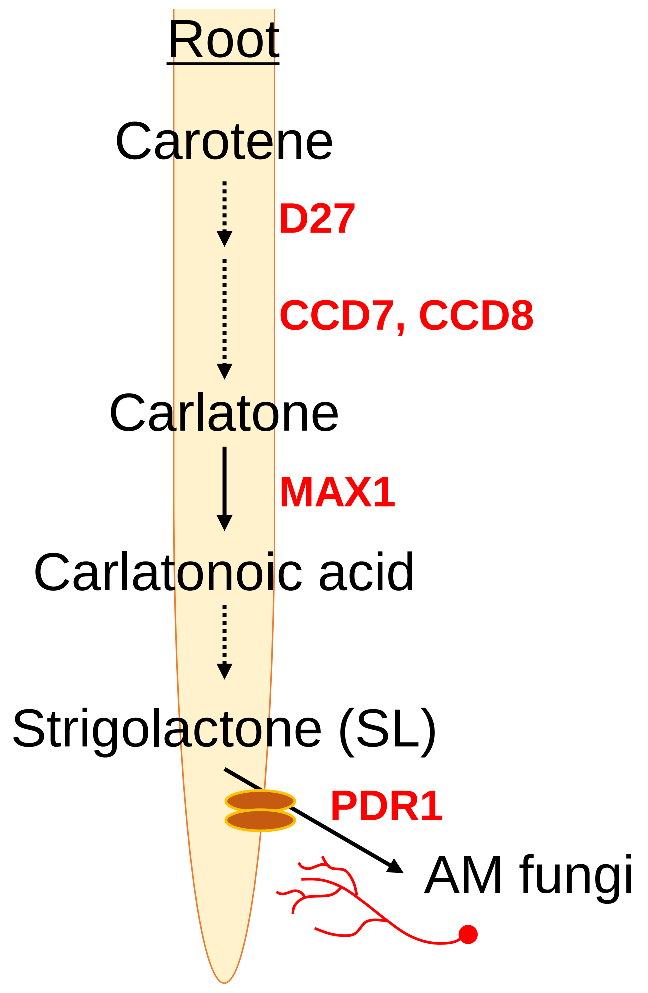

Supplementary Figure 2 | Identification of conserved AM symbiosis- and SL-related genes in host plants. **(A)** A table showing AM symbiosis-related downstream genes in *L. japonicus*, *D. carota*, and *E. grandiflorum* identified using the SonicParanoid. **(B)** A table showing SL-related downstream genes in *L. japonicus* and *D. carota*, and transcripts in *E. grandiflorum* identified using the SonicParanoid as demonstrated in **(A)** (Output of SonicParanoid: **Supplementary Tables 2, 3**). The right image illustrates the biosynthetic process and exudation of SLs in the root. Green marked areas represent the conservation of respective genes, and the values show the number of homologous genes and transcripts in *L. japonicus*/*D. carota* genomes and the *de novo* assemble data of *E. grandiflorum*, respectively (**Supplementary Table 3**).
