# Supplementary material for: Conservation and Diversity in Gibberellin-Mediated Transcriptional Responses Among Host Plants Forming Distinct Arbuscular Mycorrhizal Morphotypes
Source: Front Plant Sci. 2021 Dec 16;12:795695. doi: 10.3389/fpls.2021.795695 (PMC8718060; doi:10.3389/fpls.2021.795695)
Supplement: Supplementary file 10 [file Presentation_3.PDF]

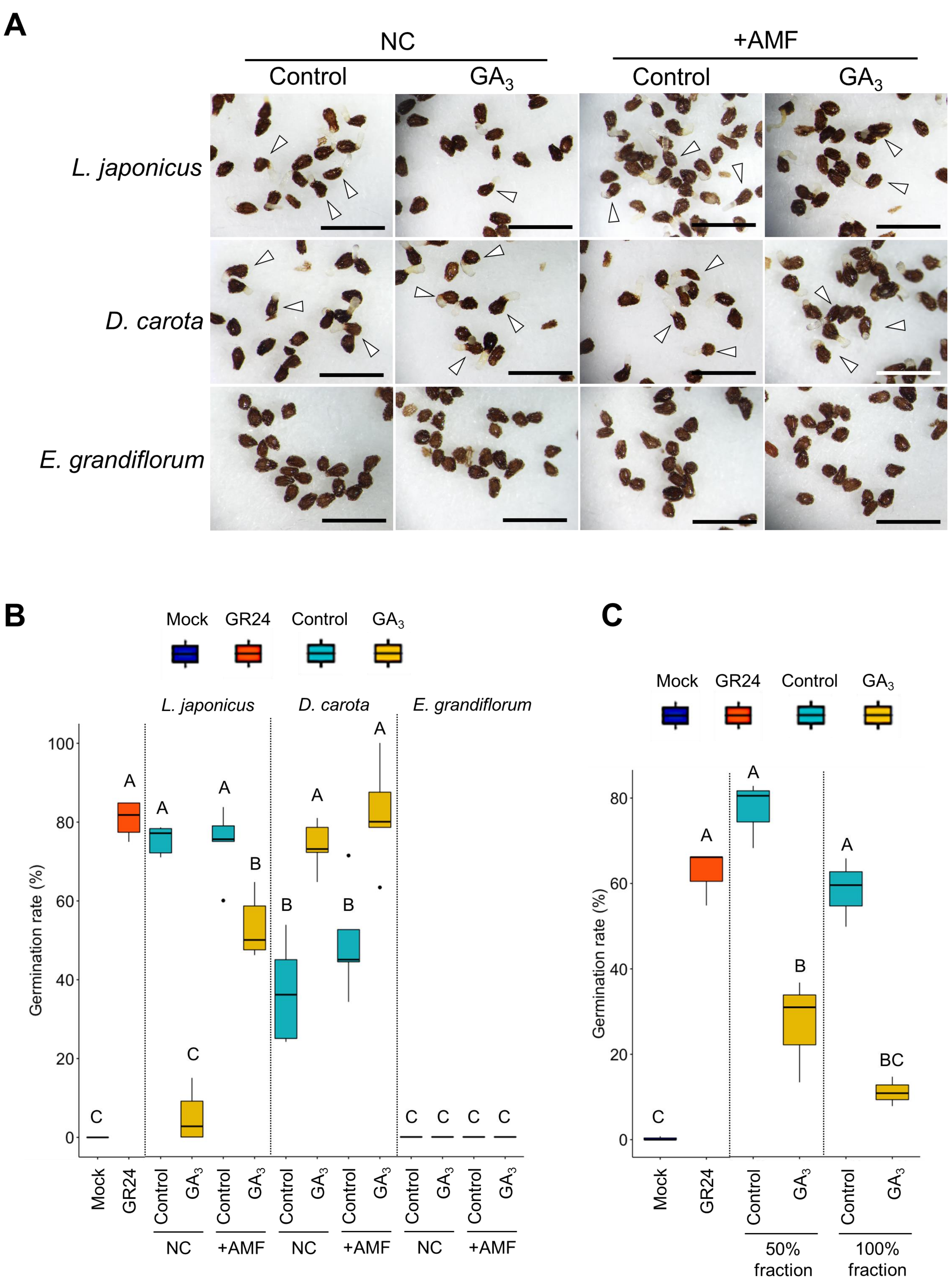

Supplementary Figure 3 | Germination rate of *O. minor* treated with the root extracts of the examined host plants. The root extracts from 6-week-old *L. japonicus*, *D. carota*, and *E. grandiflorum* roots were treated to *O. minor* seeds for 5 days. The host plants were grown under axenic (NC) and monoxenic conditions with *R. irregularis* (+AMF) in the absence and presence of 1 µM GA_3. Acetone (mock) and 1 µM *rac*-GR24 were applied for negative and positive controls, respectively. **(A)** Images of *O. minor* seeds treated with each sample. Arrowheads indicate germinating *O. minor* seeds. Scale bars, 1 mm. **(B)** Germination rate (%) of *O. minor* treated with each sample. **(C)** Germination activity of root exudates collected from 4-week-old *E. grandiflorum*. The seedlings were cultured for 2 days in distilled water with or without 1 µM GA_3, followed by reverse phase chromatography. Each fraction was obtained with 50 and 100% acetone. Different alphabets represent significant differences at $P < 0.05$ in the Tukey test (n = 3–5).
