# Supplementary material for: Conservation and Diversity in Gibberellin-Mediated Transcriptional Responses Among Host Plants Forming Distinct Arbuscular Mycorrhizal Morphotypes
Source: Front Plant Sci. 2021 Dec 16;12:795695. doi: 10.3389/fpls.2021.795695 (PMC8718060; doi:10.3389/fpls.2021.795695)
Supplement: Supplementary file 11 [file Presentation_4.PDF]

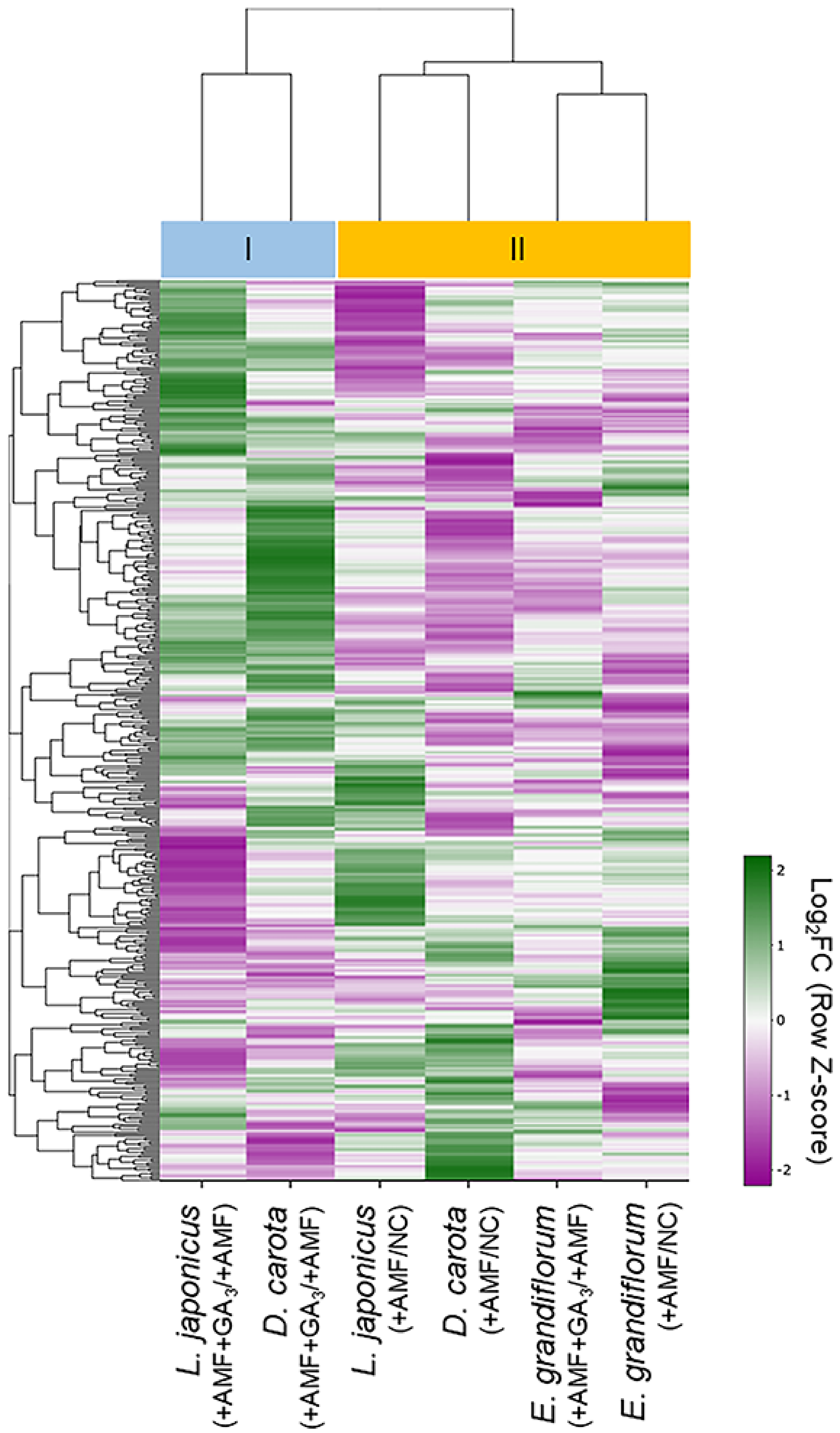

Supplementary Figure 4 | Transcriptional pattern of orthologs in response to AM fungal colonization and GA treatment. Hierarchical clustering shows the Log_2FC of the total orthologs (467 genes) compared with the controls. Green represents positive values, magenta indicates negative values, and white means zero. NC, axenic conditions; +AMF, *R. irregularis* inoculation, +AMF+GA_3, simultaneous application of *R. irregularis* inoculation and 1 µM GA_3. The DEGs were identified by comparing +AMF with NC (+AMF/NC), +AMF+GA_3 against NC or +AMF (+AMF+GA_3/NC, +AMF+GA_3/+AMF). The expression patterns of the samples were classified into two clusters, I and II. For the TPM, Log_2FC, and FDR values of the selected genes, see **Supplementary Table 5**.
