# Supplementary material for: Conservation and Diversity in Gibberellin-Mediated Transcriptional Responses Among Host Plants Forming Distinct Arbuscular Mycorrhizal Morphotypes
Source: Front Plant Sci. 2021 Dec 16;12:795695. doi: 10.3389/fpls.2021.795695 (PMC8718060; doi:10.3389/fpls.2021.795695)
Supplement: Supplementary file 12 [file Presentation_5.PDF]

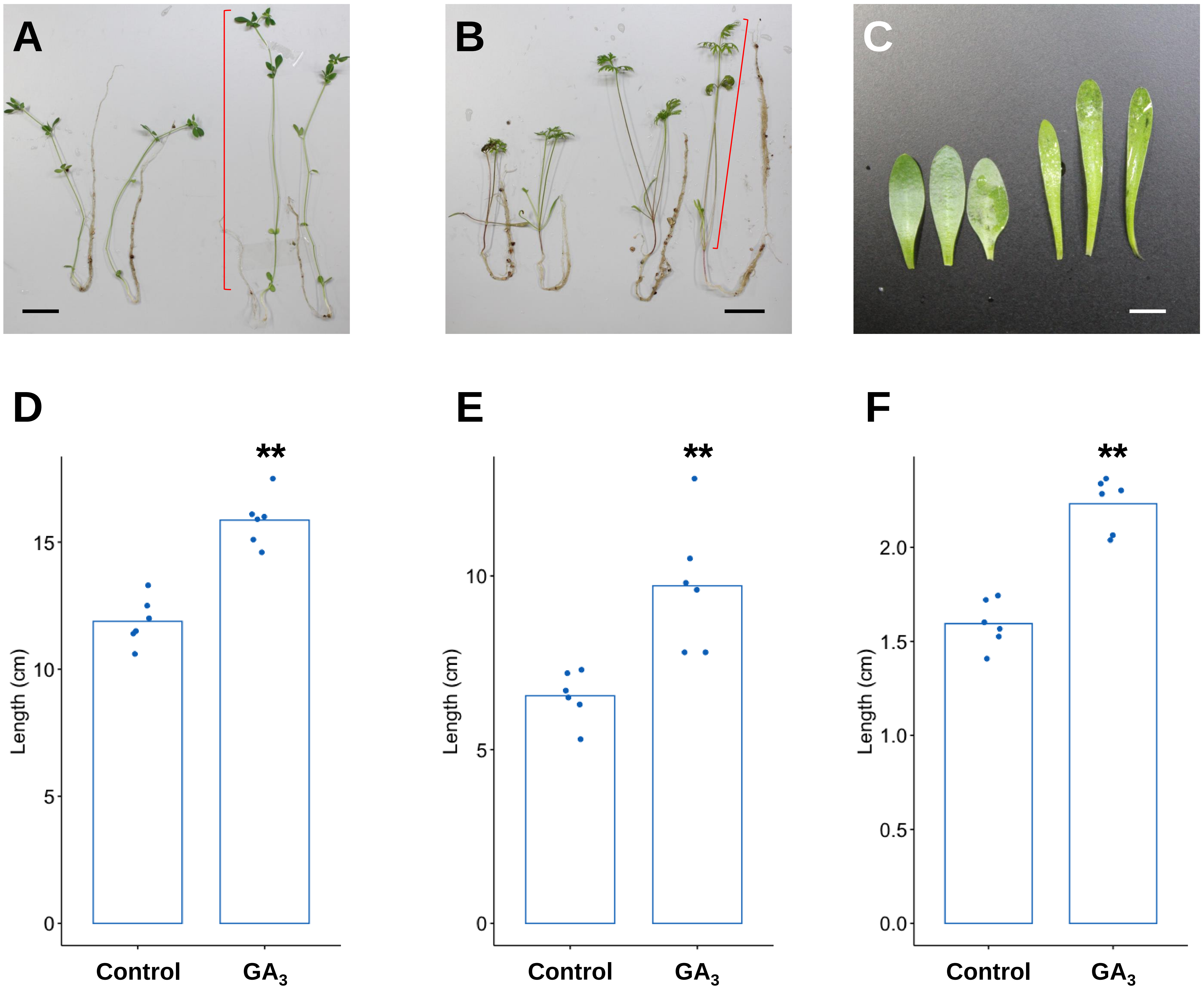

Supplementary Figure 5 | Phenotyping of GA-treated host plants. The length of *L. japonicus* and *D. carota* shoots or *E. grandiflorum* leaves were measured. These plants were treated with 0.01% ethanol as for the control and 1 μM GA_3 and grown for 6 weeks. **(A–C)** Images of GA-treated seedlings of *L. japonicus* **(A)**, *D. carota* **(B)**, or third leaves of *E. grandiflorum* **(C)**. Bars, 2 cm in **(A, B)** and 5 mm in **(C)**. **(D–F)** The length of *L. japonicus* shoot **(D)**, *D. carota* petiole **(E)**, and *E. grandiflorum* leaf **(F)**. Bars and plots indicate the mean and individual values, respectively. Asterisks show significant differences in Wilcoxon rank-sum test (**: $P < 0.01$).
