# Supplementary material for: Conservation and Diversity in Gibberellin-Mediated Transcriptional Responses Among Host Plants Forming Distinct Arbuscular Mycorrhizal Morphotypes
Source: Front Plant Sci. 2021 Dec 16;12:795695. doi: 10.3389/fpls.2021.795695 (PMC8718060; doi:10.3389/fpls.2021.795695)
Supplement: Supplementary file 13 [file Presentation_6.PDF]

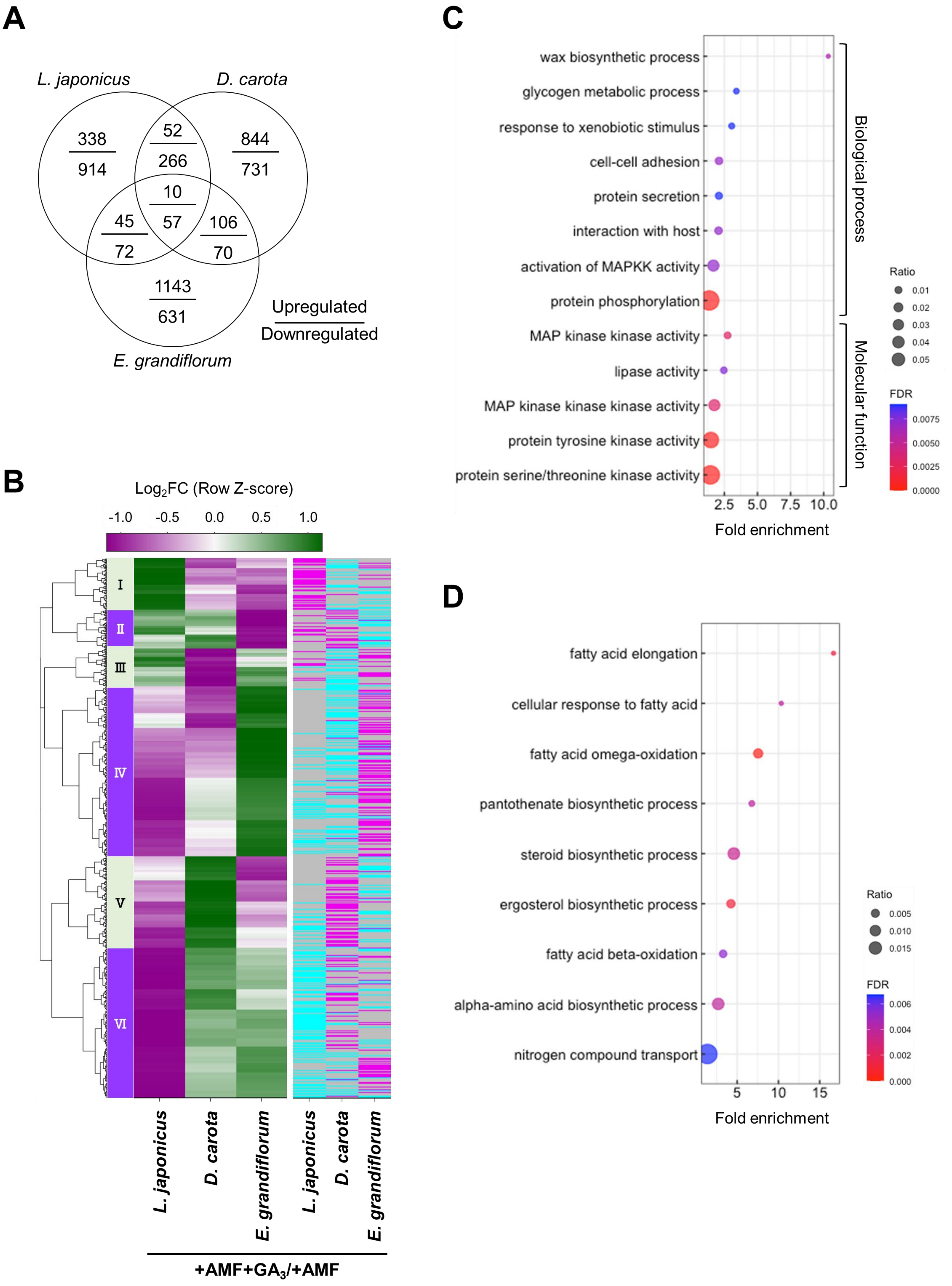
A
L. japonicus
D. carota
E. grandiflorum
338
914
52
266
844
731
10
57
45
72
106
70
1143
631
Upregulated
Downregulated
B
Log2FC (Row Z-score)
-1.0
-0.5
0.0
0.5
1.0
I
II
III
IV
V
VI
L. japonicus
D. carota
E. grandiflorum
L. japonicus
D. carota
E. grandiflorum
+AMF+GA3/+AMF
C
wax biosynthetic process
glycogen metabolic process
response to xenobiotic stimulus
cell-cell adhesion
protein secretion
interaction with host
activation of MAPKK activity
protein phosphorylation
MAP kinase kinase activity
lipase activity
MAP kinase kinase kinase activity
protein tyrosine kinase activity
protein serine/threonine kinase activity
Biological process
Molecular function
2.5
5.0
7.5
10.0
Fold enrichment
Ratio
0.01
0.02
0.03
0.04
0.05
FDR
0.0075
0.0050
0.0025
0.0000
D
fatty acid elongation
cellular response to fatty acid
fatty acid omega-oxidation
pantothenate biosynthetic process
steroid biosynthetic process
ergosterol biosynthetic process
fatty acid beta-oxidation
alpha-amino acid biosynthetic process
nitrogen compound transport
5
10
15
Fold enrichment
Ratio
0.005
0.010
0.015
FDR
0.006
0.004
0.002
0.000

Supplementary Figure 6 | Transcriptional responses to GA-treated host plants in *R. irregularis*. The *R. irregularis*-derived reads obtained from total reads of the host plants were subjected to transcriptomic analysis. The ratio of AM fungal reads is summarized in **Supplementary Table 1**. **(A)** Identification of DEGs ($|Log_2FC| > 1$, FDR < 0.05) in *R. irregularis* colonizing 1 μM GA_3-treated *L. japonicus*, *D. carota*, and *E. grandiflorum* compared when it infected the 0.01% ethanol-treated (control) plants at 6 wpi. The values represent the number of DEGs upregulated or downregulated by GA treatment. **(B)** Heatmap showing the hierarchical clustering of AM fungal genes that significantly expressed in at least one *R. irregularis* sample associating with GA-treated host plant compared with the respective control conditions (4764 genes). The Z-score-normalized Log_2FC are arranged by their expression patterns. Magenta indicates negative values, green represents positive values, and white means zero. In the right heatmap, pink and cyan show upregulated and downregulated DEGs. **(C, D)** Representative GO terms (adjusted *P*-value < 0.01) that significantly enriched in the Clusters IV **(C)** and that showing enriched biological processes in the Cluster VI **(D)**. Fold enrichment indicates the relative number of genes practically mapped to a GO term compared to the predicted number in total DEGs. The size of each circle represents ratio of DEGs enriched in a GO term to total number of DEGs. The color bar shows color-coded corrected *P*-value. *P*-values were calculated using the Fisher’s exact test and adjusted by the Benjamini–Hochberg method.
